# A critical review and weight of evidence approach for assessing the bioaccumulation of phenanthrene in aquatic environments

**DOI:** 10.1002/ieam.4401

**Published:** 2021-03-22

**Authors:** James M. Armitage, Liisa Toose, Louise Camenzuli, Aaron D. Redman, Tom F. Parkerton, David Saunders, James Wheeler, Alberto Martin, Eleni Vaiopoulou, Jon A. Arnot

**Affiliations:** ^1^ ARC Arnot Research and Consulting Inc. Toronto Ontario Canada; ^2^ AES Armitage Environmental Science Inc. Ottawa Ontario Canada; ^3^ ExxonMobil Petroleum & Chemical B.V. Machelen Belgium; ^4^ ExxonMobil Biomedical Sciences Inc. Spring Texas USA; ^5^ Shell Health, Shell International B.V. The Hague the Netherlands; ^6^ Member of Concawe Brussels Belgium; ^7^ Concawe Brussels Belgium; ^8^ Department of Physical and Environmental Sciences University of Toronto Scarborough Toronto Ontario Canada; ^9^ Department of Pharmacology and Toxicology University of Toronto Toronto Ontario Canada

**Keywords:** Bioaccumulation, Weight of evidence, Biotransformation, Phenanthrene, Biomagnification

## Abstract

Bioaccumulation (B) assessment is challenging because there are various B‐metrics from laboratory and field studies, multiple criteria and thresholds for classifying bioaccumulative (B), very bioaccumulative (vB), and not bioaccumulative (nB) chemicals, as well as inherent variability and uncertainty in the data. These challenges can be met using a weight of evidence (WoE) approach. The Bioaccumulation Assessment Tool (BAT) provides a transparent WoE assessment framework that follows Organisation for Economic Co‐operation and Development (OECD) principles for performing a WoE analysis. The BAT guides an evaluator through the process of data collection, generation, evaluation, and integration of various lines of evidence (LoE) (i.e., B‐metrics) to inform decision‐making. Phenanthrene (PHE) is a naturally occurring chemical for which extensive B and toxicokinetics data are available. A B assessment for PHE using the BAT is described that includes a critical evaluation of 74 measured in vivo LoE for fish and invertebrate species from laboratory and field studies. The number of LoE are reasonably well balanced across taxa (i.e., fish and invertebrates) and the different B‐metrics. Additionally, in silico and in vitro biotransformation rate estimates and corresponding model‐predicted B‐metrics are included as corroborating evidence. Application of the BAT provides a consistent, coherent, and scientifically defensible WoE evaluation to conclude that PHE is not bioaccumulative (nB) because the overwhelming majority of the bioconcentration, bioaccumulation, and biomagnification metrics for both fish and invertebrates are below regulatory thresholds. An analysis of the relevant data using fugacity ratios is also provided, showing that PHE does not biomagnify in aquatic food webs. The critical review identifies recommendations to increase the consistency of B assessments, such as improved standardization of B testing guidelines, data reporting requirements for invertebrate studies, and consideration of temperature and salinity effects on certain B‐metrics. *Integr Environ Assess Manag* 2021;17:911–925. © 2021 Concawe. *Integrated Environmental Assessment and Management* published by Wiley Periodicals LLC on behalf of Society of Environmental Toxicology & Chemistry (SETAC)

## KEY POINTS

- The Bioaccumulation Assessment Tool (BAT) provides a transparent framework that follows Organisation for Economic Co‐operation and Development (OECD) principles for performing a weight of evidence approach.
- A critical review of bioaccumulation and toxicokinetic data for phenanthrene is described that includes a critical evaluation of 74 measurements in fish and invertebrate species from laboratory and field studies.
- The overwhelming majority of bioaccumulation metrics are below regulatory thresholds.
- Recommendations to improve the consistency of bioaccumulation assessments are provided.

## INTRODUCTION

Chemicals are subject to bioaccumulation (B) assessment worldwide under different regulations using various lines of evidence (LoE), methods, metrics, and categorization criteria (Government of Canada 1999; UNEP 2001; EC 2007; Moermond et al. 2012; Abelkop et al. 2013). Comparing an LoE against the appropriate criterion results in a B assessment categorization outcome such as “not bioaccumulative” (nB), “bioaccumulative” (B), or “very bioaccumulative” (vB). Laboratory‐based LoE (i.e., B‐metrics) include the bioconcentration factor (BCF) and biomagnification factor (BMF) (OECD 2012). Field‐based LoE include the BMF, bioaccumulation factor (BAF), and trophic magnification factor (TMF) (Burkhard et al. 2012). In silico LoE include quantitative structure–activity relationships (QSARs) for the BCF and mass balance bioaccumulation models (e.g., Barber 2003, 2008; Arnot and Gobas 2004; Costanza et al. 2012; Mackay et al. 2013). In vitro biotransformation rate data obtained from standardized fish S9 (OECD 2018c) and hepatocyte (OECD 2018b) assays can be applied for B assessment using in vitro–in vivo extrapolation (IVIVE) methods (Nichols et al. 2013). In silico biotransformation rate data (e.g., Arnot et al. 2009; Brown et al. 2012; Papa et al. 2014; Mansouri et al. 2018), can also be considered. Despite the development of standardized testing methods (OECD 2012), in vivo laboratory B‐metrics are uncertain as a result of experimental errors and incomplete reporting of key parameters (Arnot and Gobas 2006; Barber 2008; Parkerton et al. 2008; Arnot and Quinn 2015). Field metrics are uncertain due to practical limitations with sample size (statistical power), incomplete knowledge of trophic interactions and study documentation (Borgå et al. 2012), and in some cases limits of detection (LOD) and quantification (LOQ) (Houde et al. 2008). Laboratory and field B‐metrics are also inherently variable (e.g., Burkhard 2003; Wassenaar et al. 2020), due to variability of measurements in tissue and exposure media (e.g., water, diet), differences in system parameters (e.g., temperature, pH, salinity, food web structure, sediment–water disequilibrium), and biology (e.g., lipid content, respiration, ingestion, and growth rates).

Data relevance and reliability need to be considered to establish confidence in a chemical assessment. A weight of evidence (WoE) approach (Weed 2005; Hope and Clarkson 2014; OECD 2019; Suter et al. 2020) can inform B assessment decision‐making given the various metrics and criteria and inherent variability and uncertainty in the underlying data (Barber 2003; Burkhard 2003, 2008; Arnot and Gobas 2006; Parkerton et al. 2008; Crookes and Brooke 2010; Borgå et al. 2012; Burkhard et al. 2012, 2013; Arnot and Quinn 2015; Gobas et al. 2020; Wassenaar et al. 2020). Indeed, a WoE approach for B assessment is recommended under Annex XIII of the Registration, Evaluation, Authorisation and Restriction of Chemicals (REACH) legislation in the European Union (EC 2011; Moermond et al. 2012).

Phenanthrene is a naturally occurring polycyclic aromatic hydrocarbon (PAH) that enters the environment from natural and anthropogenic combustion and petrogenic sources. Natural sources include volcanoes, forest and prairie fires, and seeps from geologic formations (Manoli and Samara 1999; Transportation Research Board National Research Council 2003). In December 2018, the European Chemicals Agency (ECHA) conducted a B assessment of phenanthrene (PHE; CAS RN 85‐01‐8) (ECHA 2018). Multiple B and toxicokinetic (TK) studies have been conducted for PHE. The ECHA B assessment relied primarily on the data compiled for PHE as part of the coal‐tar pitch, high temperature (CTPHT) assessment (ECHA 2009). The ECHA decision that PHE is “very bioaccumulative” (vB) was triggered by exceedances of the BCF criterion from a few studies with fish and invertebrate species, for example (Carlson et al. 1979; Frank et al. 1986; Jensen et al. 2012; Agersted et al. 2018). Some of the invertebrate BCF data that triggered ECHA's decision were based on lipid‐normalized chemical concentrations (i.e., BCF = (*C*_FISH_/*f*_L_)/*C*_WATER_ where *f*_L_ is the fractional lipid content), with the lipid‐normalized BCFs in the range of 40 000 to 70 000 L/kg (lipid) cited from Agersted et al. (2018) being the most notable (ECHA 2018). However, the B assessment criteria are defined on a wet weight basis (i.e., L/kg‐ww), and a large body of relevant data is available for PHE in addition to those used by ECHA (e.g., other measured BAFs and BMFs, in vitro and in silico biotransformation rates). Although the BCF takes precedence, BAFs, BMFs, and TMFs can also be considered in B assessments under REACH (ECHA 2017a). A critical review of the B and TK data and a WoE approach for assessing the bioaccumulation potential of PHE are therefore needed.

In the present study, a B assessment of PHE was conducted using the Bioaccumulation Assessment Tool (BAT) (ARC 2019). The BAT follows the guiding principles of the Organisation for Economic Co‐operation and Development WoE guidance (OECD 2019), providing a consistent and transparent framework to inform B assessment decision‐making. The BAT guides a user through the collection, evaluation, and integration of various LoE. The process of using the BAT incorporates a comprehensive and systematic evidence evaluation (data reliability scoring) to address uncertainty in multiple LoE. Summary results (evidence integration) provided by the BAT include tables and graphs of the LoE and corresponding reliability scores (RSs), the B outcome for each LoE (i.e., nB, B, or vB), fugacity ratios (Burkhard et al. 2012), and an overall strength of evidence (SoE) for a categorization outcome. The critical review of PHE B data also examines the influence of factors that can contribute significant variability to B‐metrics such as lipid content, as well as system temperature and salinity. Standardized approaches to address these sources of variability in the future are recommended.

## METHODS

### Weight of evidence approach and guiding principles

The OECD has developed formal guidance for applying a WoE approach for chemical assessments (OECD 2019). A WoE approach is a method for decision‐making that includes 5 primary elements, namely, 1) problem formulation, 2) evidence collection, 3) evidence evaluation (determine data reliability, uncertainty, and relevance), 4) evidence weighing (assign weight to evidence), and 5) evidence integration (examine evidence coherence and impact of uncertainty) (OECD 2019). Underlying these elements are the guiding principles for WoE, which include 1) a hypothesis (statement of the problem or question); 2) systematic methods (i.e., comprehensive design in data collection, evaluation, and weighting); 3) treatment of uncertainty; 4) consideration for potential bias during data collection, evaluation, and weighting; and 5) transparency by documenting the information so it can be understood and reproduced by the various stakeholders (OECD 2019). Practical implementation of a WoE approach involves consideration of known and relevant LoE where a “weight” is assigned to each LoE based on the confidence (reliability) associated with the evidence (OECD 2019). Relevant, evaluated evidence is then combined and the overall SoE is determined to support or refute a hypothesis (question) posed at the initial problem formulation stage (OECD 2019). A primary objective of WoE is to provide a transparent means for communicating decision‐making so that decisions can be clearly understood by all stakeholders (OECD 2019).

### Bioaccumulation Assessment Tool (BAT)

The BAT (Ver.1.1) is a B assessment WoE framework coded in Visual Basic for Applications (VBA) for Microsoft Excel and includes user forms and spreadsheets as a user interface (ARC 2019). The BAT includes the guiding principles and primary elements for a WoE approach recommended by the OECD (OECD 2019), as outlined above and described below. In terms of workflow for using the framework, the user is required to provide information in 4 general stages: 1) initialization/problem formulation, 2) physical–chemical properties, 3) biotransformation rate data, and 4) bioaccumulation data (in vivo, in vitro, in silico). The B‐metrics and their associated categorization thresholds (“B criteria”) are defined in the initialization/problem formulation stage; default B criteria for many regulatory programs are included, but the user is free to define their own thresholds. If a relevance weighting scheme for different B‐metrics is to be incorporated, the relative importance of the B‐metrics can also be specified using relevance weighting values ranging from 0 to 5. However, in the present study the different B‐metrics assessed were not weighted in this manner (see *Problem Formulation* section).

After physical–chemical properties are entered, the BAT guides the user through the data entry and evidence evaluation stage for each LoE using data evaluation templates (DETs). The DETs are a series of questions and criteria specific to each LoE, which assess data quality and improve transparency and consistency (reporting standards). The DETs and evidence evaluation (reliability) criteria are developed from OECD test guidelines, (e.g., OECD 2012, 2018a, 2018b, 2018c) and the peer‐reviewed literature (e.g., Klimisch et al. 1997; Arnot and Gobas 2006; Arnot et al. 2008; Borgå et al. 2012; Burkhard et al. 2013). Commentary from stakeholders informed the creation of the DETs during the development of the BAT Ver.1.0. The evaluation of data reliability is mandatory for each LoE and results in a corresponding RS. An RS from 0 to 5 is determined on the basis of information provided by the user for the questions and criteria outlined in the DETs. Most DETs include one or more criteria deemed “Critical”. If these “Critical” criteria are not met, the LoE is assigned an RS of 0 (“Critical Fail”). A Critical Fail (RS = 0) is analogous to a Klimisch score of 3 (“Not Reliable” [NR]) (Klimisch et al. 1997) as a result of the identification of major issues in the methods or reporting of the LoE during the data quality review process. The RS range of 0 to 5 provides the capacity to more explicitly differentiate the relative reliability of various LoE compared to the general Klimisch scores of 1 (“Reliable”) and 2 (“Reliable with Restrictions”) (Klimisch et al. 1997). The LoE with RS = 0 (“Not Reliable”) are documented for the sake of completeness; however, such data are not included in the WoE and SoE results. The main considerations for LoE are briefly introduced in the *Data collection, critical evaluation, and standardization* section, while complete details of the RS methods and DETs are presented in Supplemental Data Section S1.

The BAT summarizes the RS for each LoE. The B categorization results, according to the thresholds selected by the user (i.e., criteria for nB, B, or vB), for each LoE are also summarized. The BAT provides an SoE summary that refers to the frequency of bioaccumulation conclusions (i.e., nB or B or vB), based on the entered LoE and the criterion pertaining to each LoE. The higher the SoE score, the greater the number of LoE outcomes that support the same conclusion. For example, if all LoE result in an “nB” conclusion, the SoE for that chemical being judged as “nB” is 100%. This is a simple and transparent method to convey SoE for a B assessment.

The BAT uses mass balance physiologically based toxicokinetic models parameterized with the chemical partitioning and biotransformation rate data entered by the user to obtain various in silico B‐metrics (model‐calculated values). The current modeling approach in the BAT is a 1‐compartment bioaccumulation model that assumes the biotransformation rate constants apply to the whole body level, including liver, gill, gastrointestinal tract, and other tissues. The model‐calculated values provide additional LoE for comparison to empirical B‐metrics and address data gaps when empirical data are limited or not available. Consensus between simulated in silico metrics and reliable quality empirical data builds further confidence in the WoE. Significant divergence between simulated (model‐calculated) B‐metrics and reliable quality empirical data can provide guidance for addressing uncertainty and ultimately reconciling possible inconsistencies between the empirical data (e.g., unresolved error or variability) and simulations (e.g., model or input parameter error), when necessary.

### Problem formulation

In addition to the scientific challenges associated with establishing confidence in a B assessment decision, there is debate as to which B‐metrics are the most relevant (Gobas et al. 2009; Moermond et al. 2012; Matthies et al. 2016). Bioaccumulation categorization criteria in most regulatory jurisdictions are specified by metrics that relate organism to water concentrations, that is, BCFs and BAFs; however, prevailing guidance from the scientific community advocates for metrics related to biomagnification, that is, BMFs and TMFs (Gobas et al. 2009; Moermond et al. 2012; Matthies et al. 2016). The lack of scientific and regulatory consensus is addressed in the present assessment by establishing 2 different questions (hypotheses) that can be answered with a WoE approach. The first problem formulation scenario uses the wet weight BCF and BAF criteria ≥2000 L/kg (B) and ≥5000 L/kg (vB) for B categorization. The second scenario uses the lipid‐normalized BMF and TMF criterion >1 (vB) or <1 (nB) for B categorization (Gobas et al. 2009; Burkhard et al. 2012). The first scenario is consistent with the REACH screening criterion (i.e., BCF only), but not the WoE approach outlined in Annex XIII (i.e., BCF and other LoE). The second scenario reflects the recommendations from a SETAC Pellston Workshop (27 January to 1 February 2008, Pensacola, Florida, USA) entitled “Science‐Based Guidance and Framework for the Evaluation and Identification of PBTs and POPs” (i.e., TMF and BMF as the higher tier B assessment metrics) (Gobas et al. 2009). The advantage of the second scenario is that additional exposure to higher organisms through the diet, driven by biomagnification, is considered. In contrast, the BCF, particularly for invertebrate species, more closely reflects equilibrium partitioning (EQP) of the chemical between the organism and water.

### Relevant physical–chemical properties and temperature and salinity effects

Aquatic bioaccumulation is primarily a function of chemical partitioning between water, diet, and organism as well as biotransformation processes. The octanol–water partition coefficient (*K*_OW_) can be used to quantify bioconcentration potential for neutral, hydrophobic, poorly biotransformed organic chemicals (Mackay 1982) in which octanol is a surrogate for organic phases in the organism. Water solubility and partition coefficients like *K*_OW_ are a function of system temperature (Beyer et al. 2002; Schwarzenbach et al. 2003) and salinity (“salting‐out” effect) (Xie et al. 1997; Endo, Pfennigsdorff et al. 2012). Because physical–chemical properties and bioaccumulation are a function of system properties, B‐metrics obtained from different systems may require interpretation to allow appropriate comparisons. Temperature and salinity modulate B‐metrics that rely on aqueous exposure concentrations like the BCF and BAF. The temperature dependence of solubility and partitioning can be estimated using internal energies of phase change and the van't Hoff equation (Schwarzenbach et al. 2003). Adjustments for the salting‐out effect on solubility and *K*_OW_ have been established using Setschenow constants and the concentrations of various salts present in solution (Schwarzenbach et al. 2003).

The BAT requires information on various physical–chemical properties (ARC 2019). The critically evaluated property values for PHE reported by Ma et al. (2010) in fresh water at 25 °C have been selected and are summarized in the Supplemental Data (Section S2, Table S2‐1). Experimental data for PHE indicate that the sorption capacity of surrogate biological lipids (e.g., fish oil, olive oil, milk fat) is greater than that of octanol (Mayer et al. 2009; Geisler et al. 2011); therefore, BAT was also parameterized with partition coefficients for storage lipids, membrane lipids, serum albumin, and structural proteins estimated using poly‐parameter linear free energy relationships (ppLFERs) (Endo and Goss 2014). The ppLFERs generate biopartition coefficients at 37 °C and were corrected to 25 °C using an internal energy of phase change (Schwarzenbach et al. 2003; Geisler et al. 2012) (Supplemental Data Table S2‐1). The estimated internal energy of phase change (–20.3 kJ/mol) corresponds to a 1.3‐fold increase in the biopartition coefficient for every 10 °C decrease in temperature. The estimated storage lipid–water partition coefficient at 25 °C is 4.81, which is 0.34 log units (i.e., approximately 2‐fold) greater than *K*_OW_ (log *K*_OW_ = 4.47) and is consistent with available experimental data. Details of the underlying partition coefficient calculations are provided in the Supplemental Data. Supplemental Data Table S2‐1 also includes internal energies of phase change reported by Beyer et al. (2002) for *K*_OW_, and air–water and octanol–air partitioning (*K*_AW_, *K*_OA_, respectively). Temperature dependence is included in the calculation of fugacity ratios and the BAT in silico B‐metrics described in the *Application of equilibrium criterion to B data* section.

The Setschenow constant for PHE was estimated based on the approach suggested by Ni and Yalkowsky (2003) and corresponds to a decrease in water solubility in seawater (NaCl at 0.5 M) of a factor of 1.4. All else being equal, partition coefficients between octanol, lipids, and seawater will increase by the same factor (e.g., *K*_OW_ is 1.4‐fold greater in seawater vs fresh water) (Xie et al. 1997). Temperature dependence and salinity are not explicitly considered to adjust any user‐entered LoE within BAT Ver.1.1; however, when relevant in the present assessment, the measured B data are examined for the influence of temperature and salinity “manually” outside of the BAT application.

### Data collection, critical evaluation, and standardization

The PHE B and TK data collected for the ECHA regulatory decision (ECHA 2018) as well as reviews generated by other organizations (e.g., Bleeker and Verbruggen 2009; Verbruggen 2012) were considered. Additional in vivo data from the peer‐reviewed literature were also included. Study details for measured BCFs, BAFs, BMFs, and TMFs are provided in the Supplemental Data. All BCF, BAF, and BMF data were compiled as originally reported and then converted to wet weight values as detailed in the Supplemental Data. General considerations for data quality and standardization are summarized in the following paragraphs.

The regulatory criteria for BCFs and BAFs are for wet weight values with units L/kg‐ww; however, there are studies in which these metrics are reported on a dry weight (dw) or lipid weight (lw) basis. The BMFs are typically reported on a lipid weight basis (i.e., lipid‐normalized) so the change in fugacity between the prey and the predator is more clearly conveyed, but there can be exceptions (e.g., wet weight basis or additionally normalized to trophic level). According to OECD 305 guidance for fish bioaccumulation testing (OECD 2012), wet weight BCFs on a “5% lipid normalized” basis are recommended for B assessment to address differences between fish lipid contents. For clarification, the “5% lipid normalization” terminology in OECD 305 (OECD 2012) differs from the terminology of “5% lipid standardization” used here. The latter terminology is recommended because 1) the process aims to standardize wet weight BCFs (i.e., L/kg‐ww) to a common whole body lipid content to address variability that often occurs due to lipid variability in fish and tests, and 2) to avoid any possible confusion with the process of lipid normalization, which has been used for decades to express biota concentrations and B‐metrics on a lipid weight basis (i.e., L/kg‐lw).

There are 3 key assumptions inherent to using a 5% lipid standardization for fish BCFs. The first is that lipids dominate the total sorption capacity for a chemical in an organism (Mackay 1982). The first assumption is generally approximated for hydrophobic neutral organic chemicals; however, for organisms and tissues with low lipid contents (<~2%), it has long been understood that other phases (e.g., proteins) require consideration (Arnot and Gobas 2004; Mackintosh et al. 2004; deBruyn and Gobas 2007; Endo, Bauerfeind et al. 2012; Mäenpää et al. 2015). When nonlipid phases contribute significantly to the sorptive capacity of the organism, the “lipid‐equivalent” standardization methods are more appropriate (Mackintosh et al. 2004; deBruyn and Gobas 2007; Mäenpää et al. 2015). The second assumption is that BCFs and BAFs for all chemicals scale proportionally (1:1) with lipid content. This assumption is valid only if passive elimination processes dominate the overall bioaccumulation process such that biota concentrations (*C*_B_) approach equilibrium values with the concentration in water (i.e., *C*_B_ = *K*_BW_ · *C*_W_, where *K*_BW_ is the biota–water partition coefficient). A review of the BCF and BAF data for PHE in fish and invertebrates indicates that this assumption is met for only a few B‐metrics from invertebrates. The third assumption is the selection of an appropriate median value for standardization of lipid contents. A 5% lipid content for fish is a reasonable value, given that extensive reviews of existing BCF (Arnot and Gobas 2006) and BMF (Arnot and Quinn 2015) data for fish show a central tendency for lipid contents of approximately 5%. For invertebrates, it is not clear what whole body lipid content is representative of central tendency for standardization.

To address the potential contribution of nonlipid phases to the total sorption capacity of lean organisms and avoid the bias introduced by using a “lipid only” standardization method, the B assessment for PHE is conducted using both wet weight BCF and BAF values and 5% lipid‐equivalent standardized wet weight BCF and BAF values for fish and invertebrates. The following approach for lipid‐equivalent standardization was applied, shown here for a wet weight BCF (*BCF*_*WW*_):

(1) $$\mathrm{BCF}5\%\mathrm{EQ}={\mathrm{BCF}}_{\mathrm{WW}}\;\cdot \;\frac{0.05}{{(f}_{L}+\varphi f_{P})},$$

where *f*_L_ is the total lipid content, *f*_P_ is the structural protein content of the organism, and *ϕ* is the proportionality between the protein and lipid–water partition coefficients (i.e., *ϕ* = *K*_PW_/*K*_SLW_ where *K*_PW_ and *K*_SLW_ are protein–water and storage lipid–water partition coefficients, respectively). See Supplemental Data Section S3 for more details. Protein contents of aquatic organisms are rarely reported in bioaccumulation studies but typically range from 10% to 20% on a wet weight basis (van der Meeren et al. 2008; Breck 2014; Mäenpää et al. 2015; Tabakaeva et al. 2018) and a value of 15% was assumed for all standardizations herein. Methods for calculating lipid standardized and lipid‐equivalent standardized B‐metrics are compared in Supplemental Data Section S3. The sensitivity of 5% lipid‐equivalent standardized wet weight BCFs to protein content is also documented in Supplemental Data Section S3 and, as expected, is relatively small for organisms with lipid contents greater than 2%.

#### Fish data

The importance of fish B data for B assessment is well recognized (Arnot and Gobas 2006; Moermond et al. 2012; ECHA 2017b), and an OECD guideline for testing, reporting, and interpretation has been published for aqueous and dietary laboratory exposures (OECD 2012). Fish data compiled for the present assessment are documented in Supplemental Data Section S4. Key issues for assessing data reliability have also been published (e.g., Arnot and Gobas 2006; Parkerton et al. 2008) and include 1) unambiguous reporting of units, 2) appropriate exposure conditions (stable concentrations, below water solubility and toxicity thresholds), and 3) direct measurement of chemical in water and test organisms. Primary considerations for assessing the reliability of field B data (BAFs, BMFs) include analytical quality, for example, use of blanks and standards, reporting of LOQ, adequate and appropriate sampling design, and reporting of ancillary data, for example, lipid contents. See the DETs in Supplemental Data Section S1 for further details about how RS are determined. Given the scope of current testing guidance (OECD 2012), only studies with juvenile or adult fish were considered in the BAT. The BCFs for fish were 5% lipid‐equivalent standardized where required data were available, but no adjustments were made to account for the influence of temperature and salinity differences on the magnitude of the B‐metrics.

#### Invertebrate data

Although measured B data from fish obtained from standardized testing guidelines (OECD 2012) are historically considered for decision‐making, invertebrate B data were also considered by ECHA for the PHE B assessment (ECHA 2018). Exposure conditions for the invertebrate studies were more variable than the fish data, that is, water temperatures ranged from 2 to 25 °C, and B‐metrics were reported in various units, that is, BCFs on dry, wet, or lipid basis. Ancillary data necessary to standardize the data for comparative purposes were less frequently provided. This reflects the fact that protocols and reporting standards for invertebrate BCF test data on par with the OECD 305 guideline for fish are still under development (e.g., Schlechtriem et al. 2019). The RS in BAT Ver.1.1 for the invertebrate data were derived using the same DETs used to score fish studies. Based on available data and assumptions, the invertebrate BCF data were calculated on a wet weight basis and 5% lipid‐equivalent standardized values to be consistent with the treatment of the fish data (see Supplemental Data Section S5 for details). The assumption of using 5% lipid‐equivalent standardization for invertebrates is considered conservative because invertebrates in the environment typically have lower whole body lipid contents than fish. Whether or not using 5% lipid as a standardized value for invertebrates is appropriate requires further consideration and consensus guidance, but that is beyond the scope of the present work. As with the fish data, there are no adjustments of empirical B data to account for temperature and salinity in the BAT.

#### Trophic magnification factor data

Trophic magnification factors represent the average factor change in biota concentrations as a function of trophic level in a food web (Borgå et al. 2012; Conder et al. 2012). Trophic magnification factors calculated from lipid‐normalized concentrations greater than 1 indicate biomagnification, whereas TMFs less than 1 indicate biodilution (Gobas et al. 2009; Borgå et al. 2012; Conder et al. 2012). Reliability scores for TMFs are based primarily on issues regarding analytical quality (use of field blanks, appropriate standards, reporting of LOQs), trophic level assignment, and representativeness of sampling (number of samples, range of trophic level, contemporaneous collection) (Supplemental Data Section S1). A critical consideration for TMF data is the *p*‐value of the slope of the regression between concentration and trophic level, which indicates whether the slope is significantly different from 0 and the TMF is significantly different from unity. Slopes with *p*‐values greater than 0.05 are not statistically different from 0 and hence are inconclusive regarding concentration trends with trophic level. Such studies provide no evidence of trophic magnification or dilution regardless of the TMF value. Although not deemed “Critical Fails,” we decided to exclude all TMFs with *p*‐values > 0.05 from the final SoE.

#### Biotransformation rate and in silico data (BAT‐predicted B‐metrics)

We compiled in vitro biotransformation rates (Nichols et al. 2018, 2019), in vivo whole body biotransformation rate estimates (*k*_B_) (Arnot et al. 2008), and applied OECD‐validated QSARs to generate whole body biotransformation half‐lives (HL_B_) in fish (Arnot et al. 2009; Brown et al. 2012; Papa et al. 2014; Mansouri et al. 2018). The HL_B_ data are used as input parameters for the BAT in silico B‐metric calculations, which can then be compared to the empirical data to support the WoE. Based on chemical property and biotransformation rate data entered by the user, the BAT calculates the following B‐metrics for fish using a mechanistic mass balance modeling approach: 1) laboratory BCF (5% lipid standardized fish), 2) field BAF (low trophic‐level fish), 3) laboratory BMF, and 4) field BMF (same low trophic‐level fish). Details on the bioaccumulation modeling and other assumptions required for the calculations (e.g., laboratory test conditions, environmental conditions, organism properties, default toxicokinetic parameters) are detailed in the BAT user guidance document (ARC 2019). Model predictions in good agreement with observations are valuable because they demonstrate concordance between the expected behavior of a chemical, given its partitioning properties, and biotransformation rate data and hence provide additional confidence in the decision‐making process.

#### Application of equilibrium criterion to B data

Burkhard et al. (2012) outlined an approach to facilitate the interpretation and comparison of laboratory and field B data using fugacity ratios (Mackay 2001). Fugacity (*f*) is an equilibrium criterion calculated from the concentration (*C*) in each medium and the sorption (storage) capacity of that medium (*Z*), that is, *f* = *C*/*Z*, where *Z* is a function of phase composition (e.g., lipid content, protein content, water content) and partitioning data. Fugacity ratios between 2 phases equal to 1 indicate that the chemical has achieved thermodynamic equilibrium (i.e., equivalent chemical potential or activity). Fugacity ratios are thus concentration ratios normalized to sorption capacity, and in the case of BCF data, can also be understood as ratios versus EQP–based values, that is,

(2) $$\mathrm{Fugacity}\;\mathrm{Ratio}=\frac{f_{\mathrm{BIOTA}}}{f_{\mathrm{WATER}}}=\frac{C_{\mathrm{BIOTA}}}{C_{\mathrm{WATER}}}\;\cdot \;\frac{Z_{\mathrm{WATER}}}{Z_{\mathrm{BIOTA}}}=\frac{\mathrm{BCF}}{K_{\mathrm{BW}}},$$

where *K*_BW_ is the equilibrium biota–water partition coefficient (i.e., *Z*_BIOTA_/*Z*_WATER_).

The BAT calculates fugacity ratios for BCF, BAF, BMF, and TMF data entered by the user, if required inputs are provided. Based on theoretical considerations (Mackay 2001; Burkhard et al. 2012), BCFs are expected to exhibit fugacity ratios (*f*_BIOTA_/*f*_WATER_) equal to or less than 1 because bioconcentration is driven by organism–water partitioning. Fugacity ratios less than 1 are often the result of biotransformation occurring in the organism. Assuming body composition (e.g., lipid content) and partitioning data are accurate, fugacity ratios greater than 1 for BCFs imply error in the reported concentrations in the organism, water, or both.

Biomagnification factors with fugacity ratios greater than 1 indicate biomagnification, whereas BMFs with fugacity ratios less than 1 indicate biodilution (Burkhard et al. 2012). Fugacity ratios greater than 1 are possible for BAFs and indicate that dietary uptake is important (i.e., biomagnification is occurring). The extent to which a BAF fugacity ratio exceeds 1 is a function of chemical and organism properties, food web characteristics, environmental conditions, and trophic level. However, any conclusion drawn from a BAF regarding biomagnification should be consistent with the BMF for the same organism. For example, BAF fugacity ratios much greater than 1 (biomagnification) cannot be reconciled with BMF fugacity ratios much less than 1 (biodilution). In other words, fugacity ratios for BMFs and BAFs determined under the same conditions in the same organism should be consistent.

Empirical BCFs with fugacity ratios greater than 1 are not automatically excluded from the B assessment in the current version of the BAT, nor are discrepancies between BAF and BMF fugacity ratios “flagged” for the user. However, the fugacity ratios should always be reviewed and considered as part of the interpretation and decision to include or exclude various LoE in the WoE and SoE.

## RESULTS

### Bioaccumulation data summary: BCF, BAF, BMF, TMF

All BCF, BAF, BMF, and TMF collected for the WoE assessment are presented individually in Supplemental Data Sections S4 to S5. There are 43 measured values for fish and 25 measured values for invertebrates that were critically evaluated in the present assessment. Six TMF studies were also included for a total of 74 in vivo LoE, which are reasonably well balanced across taxa (i.e., fish vs invertebrates) and the different B‐metrics used in the 2 problem formulation scenarios (i.e., BCFs and BAFs vs BMFs and TMFs). A substantial effort was required to evaluate and standardize the invertebrate BCF data because of inconsistencies in the reporting units (wet, dry, and lipid‐basis) and lack of ancillary data on organism composition (e.g., lipid and water contents) and other study details. A study reporting PHE bioaccumulation in fish eggs and larvae was found (Petersen and Kristensen 1998); however, these PHE data (see Supplemental Data Section S4, Table S4‐7) are excluded from the B assessment because toxicity was observed in all experiments with larvae (malformation; bilaterally bent chorda) and the exposure concentrations were very high (within 10% of water solubility). The exclusion of these data is consistent with 1) a previous summary of bioaccumulation data for PAHs where data from early life stages were deemed “Not Reliable” (Bleeker and Verbruggen 2009) and 2) OECD 305 testing guidelines (OECD 2012).

### Biotransformation rate data summary: In vivo, in silico, and in vitro

Biotransformation is a key process influencing the bioaccumulation potential of hydrophobic organic chemicals and is an important parameter for B model calculations. There are 4 “good” or “moderate” confidence whole body in vivo HL_B,N_ values (half‐lives normalized to 0.01 kg body size at 15 °C) for 3 species of fish (Arnot et al. 2008) ranging from 1.56 to 4.51 d (geomean = 2.16 d). The corresponding rate constants range from 0.44 to 0.15/d (geomean = 0.32/d). Three other estimates in the in vivo HL_B_ database (Arnot et al. 2008) that were classified as “low” or “uncertain” confidence were not used here. There are 6 in silico predictions for HL_B,N_ ranging from 1.39 to 4.01 d (Arnot et al. 2009; Brown et al. 2012; Papa et al. 2014; Mansouri et al. 2018); all predictions are within the defined QSAR applicability domains (OECD 2004, 2007). The geometric mean of the in silico HL_B,N_ is 2.13 d, which corresponds to a rate constant of 0.33/d. There are 2 in vitro biotransformation rate estimates that, when scaled to HL_B,N_ using IVIVE models, are 2.56 and 3.26 d (geomean = 2.89 d, rate constant = 0.24/d) (Nichols et al. 2018, 2019). The biotransformation data obtained from multiple studies show strong consistency across in vivo, in vitro, and in silico HL_B,N_ estimates for fish; the slightly longer HL_B,N_ from the in vitro assays may be due to extrahepatic biotransformation or natural variability or other factors. The estimates of central tendency and uncertainty are used in the BAT in silico model calculations (ARC 2019) to predict the average values and ranges of BCFs, BAFs, and BMFs for several organisms and 2 environments (laboratory, field).

### Reliability scoring summary: BCF, BAF, BMF, TMF

**Figure 1:**
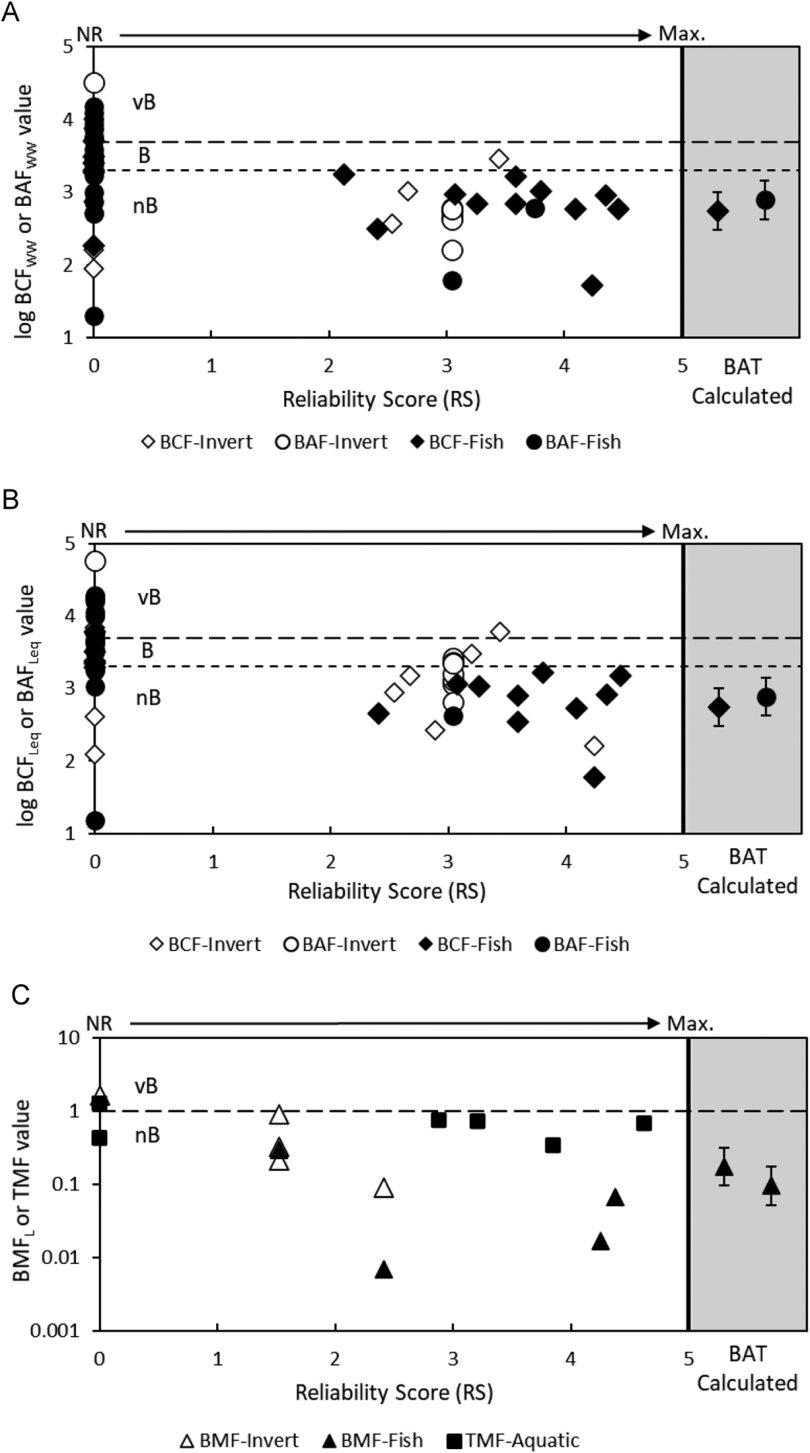
Bioaccumulation data for fish and invertebrates in comparison to “B” and “vB” threshold criteria and associated RSs from 0 (Not Reliable/Critical Fail) to 5 (Most Reliable). BCF and BAF data on a wet weight basis (**A**); BCF and BAF data on a wet weight 5% lipid‐equivalent basis (**B**); lipid‐normalized BMFs and TMF data (**C**). In silico data (i.e., BAT model calculations) are plotted in the gray section for comparative purposes and are not assigned an RS. B =bioaccumulative; BAF = bioaccumulation factor; BAT = Bioaccumulation Assessment Tool; BCF = bioconcentration factor; BMF = biomagnification factor; nB =nonbioaccumulative; NR = Not Reliable (Critical Fail); RS = reliability score; TMF = trophic magnification factor; vB = very bioaccumulative; ww = wet weight.

Reliability scores for each study are shown in Figure 1 and documented in Supplemental Data Section S6. Additional details can also be found in the BAT summary output (also provided as Supplemental Data). For some studies, more than 1 LoE (i.e., reported B metric) may be available because of different study conditions or trials (e.g., low dose, high dose). Reliability scores for fish BCFs (*n* = 17) ranged from 0 (Critical Fail, *n* = 6) to 4.46 with an average RS of 3.54 if only LoE with RS > 0 are considered. The RS of fish BAFs (*n* = 22) ranged from 0 (Critical Fail, *n* = 20) to 3.75 with an average of 3.40 if only LoE with RS > 0 are included. The lab BMFs (*n* = 2) had RS of 4.25 and 4.38, whereas the field fish BMFs (*n* = 2) had RS of 1.52 and 2.41. In many studies, information required to assess study reproducibility and analytical quality was missing. For example, the LOQ or LOD were only rarely reported, and study conditions such as water temperature and organic carbon content and fish size and lipid content were often not reported, thus lowering the RS. For all fish LoE, studies with RS > 0 reported lower B‐metrics compared to studies with RS = 0.

Reliability scores for invertebrate BCFs (*n* = 11) ranged from 0 (Critical Fail, *n* = 5) to 4.24 with an average RS of 3.16 if only LoE with RS > 0 are included. The RS of invertebrate BAFs (*n* = 8) ranged from 0 (Critical Fail, *n* = 1) to 3.04. The invertebrate field BMFs (*n* = 6) had RS of 0 (Critical Fail, *n* = 1) and 2.41. As mentioned, many invertebrate studies were lacking key ancillary data and reported BCFs or rate constants without unambiguously stating units (e.g., dry weight vs wet weight). Similar deficiencies as noted for the fish data above (e.g., analytical quality) were also common with the invertebrate studies, thus lowering RS in those instances.

Reliability scores for the 6 TMF studies ranged from 2.88 to 4.62. Deficiencies in these studies were mostly related to sampling design and reporting of information relevant to analytical quality. Two TMF studies were excluded from the final BAT WoE because the slopes of the TMF regression had *p*‐values greater than 0.05 (i.e., slope not statistically different from 0). When the slope is not statistically different from 0, no valid conclusion can be drawn regarding trophic magnification or dilution, and hence the TMF cannot be included and used to inform the SoE summary.

The most notable outcome of the RS exercise for laboratory fish BCF data is the exclusion of the BCFs from Carlson et al. (1979), which ranged from 1900 to 5100 L/kg as originally reported. The main reason for excluding these data was failure to demonstrate a reliable plateau indicating “steady‐state” in fish concentrations over the exposure period, resulting in reported BCF values that are “Not Reliable” (i.e., Critical Fail). This data quality assessment is consistent with OECD 305 guideline requirements for valid BCF calculations (OECD 2012). The exclusion of these data has important implications for B assessment because the remaining fish studies all have BCF < 2000 L/kg.

The Carlson et al. (1979) BCFs were key data used for the categorization of PHE as “vB” by ECHA (ECHA 2018). For these reasons, a detailed reanalysis of Carlson et al. BCFs was conducted as detailed in the Supplemental Data Section S7. Briefly, gill uptake (*k*_1_) and total elimination rate constants (*k*_T_) from reported water and fish concentrations were calculated using the bcmfR Tool (version 0.4‐18) (OECD 2012) along with kinetic BCFs (i.e., BCF_K_ = *k*_1_/*k*_T_). The ensuing BCF_K_ standardized to 5% lipid range from 1956 to 3826 L/kg for the 2 “phenanthrene only” tests and between 1488 to 2299 L/kg for the 3 tests in which PHE was tested in a mixture. However, due to the lack of reproducibility and uncertainty in the toxicokinetic parameters and BCF estimates between and within single compound and mixture tests, it was concluded that the precision of BCF results obtained from the Carlson et al. (1979) study were inadequate to provide a defensible basis for using these data in the present B assessment. Additionally, the method used for quantification of PHE in fish tissue based on HPLC separation followed by GC analysis using photoionization detection raises additional concerns regarding measurement accuracy given the reported variability in PHE recoveries for 8 spiked fish tissue samples that ranged from 67% to 116% (c.f. Table 8 in Carlson et al. 1979). Furthermore, evaluation of the initially reported “steady‐state BCFs” using fugacity ratios also highlights likely errors because fugacity ratios close to or exceeding 1 (i.e., the theoretical maximum) were calculated for some tests. Fugacity ratios approaching 1 imply negligible biotransformation rates, which is inconsistent with the observed rapid loss of PHE during the depuration period in all 5 tests and the aryl hydrocarbon hydrolase enzyme activity that was reported in the Carlson et al. (1979) study. Moreover, there are higher confidence (more reliable, less uncertain) fish BCF data available, and hence there is no need to rely on data that are “Not Reliable” as a key regulatory driver in the PHE B assessment (ECHA 2018). In fact, the use of data that are “Not Reliable” contradicts the guiding principles of a WoE approach (OECD 2019).

The field BAFs reported by Khairy et al. (2014) are excluded because the fugacity ratios of the reported BAFs indicate substantial biomagnification is occurring (i.e., BAF fugacity ratios > 1), whereas the TMF from the same study (RS = 3.9) indicates biodilution (TMF = 0.34, *p*‐value of slope = 0.002). As explained in the *Methods* section, such results are irreconcilable. A plausible explanation for this discrepancy is error in the reported water concentrations used to derive the BAFs (see Supplemental Data Section S8 for further discussion). Field BAFs must be judged with caution and are more difficult to use in B assessment because variation in the exposure (diet and aqueous) estimates is often greater (or unknown) compared to laboratory studies where these variables are controlled and well quantified.

### The influence of temperature and salinity on B‐metrics

As documented in the Supplemental Data, B‐metrics for fish and invertebrates include observations covering a range of water temperatures (2–25 ^o^C) and salinities (freshwater and marine). The range of water temperature corresponds to approximately a 2‐fold change in estimated partition coefficients, whereas the change in water solubility between a freshwater and a marine environment and subsequent biota–water partitioning corresponds to a 1.4‐fold change. For a 5% lipid and 15% protein content organism, the estimated EQP‐based BCF (ignoring biotransformation) of PHE in fresh water ranges from 3510 L/kg at 25 °C to 6940 L/kg at 2 °C. All else being equal, the EQP BCF for the same organism in a marine environment ranges from 4910 L/kg at 25 °C to 9715 L/kg at 2 °C. For a 2% lipid and 15% protein content organism, the EQP BCF in fresh water ranges from 1570 L/kg at 25 °C to 3110 L/kg at 2 °C and from 2200 L/kg at 25 °C to 4350 L/kg at 2 °C in a marine environment. In other words, in the absence of biotransformation (assuming PHE is perfectly persistent in biota), differences in environmental conditions appear to be sufficient to result in changes in BCF that are large enough to change B assessment decisions (e.g., nB to B, B to vB) for chemicals with partitioning properties like PHE.

For PHE, the largest BCFs and BAFs for invertebrates tended to be reported for marine conditions and cold‐water temperatures (2–7 ^o^C) (Baussant et al. 2001; Agersted et al. 2018). These observations lend support to the calculations previously described. It has been suggested that LoE used in hazard assessment should be compared to the conditions in which the hazard criteria were derived (Matthies et al. 2016). Following this logic, the 5% lipid‐equivalent BCF of 6026 L/kg for *Mytilus edilus* in seawater at 7 °C (Baussant et al. 2001) would be 1.4‐fold lower (4300 L/kg) for a mollusk in fresh water and 2.4‐fold lower (2510 L/kg) for a mollusk at approximately 25 °C in fresh water. This analysis highlights the practical challenges of interpreting BCF data and providing consistent B categorization decisions across substances in a regulatory context when B data are obtained from a range of environmental conditions.

### Evidence integration: BAT summary

Figure 1 summarizes the LoE plotted against their RS. Empirical BCFs and BAFs are presented on a wet weight (Panel A) and wet weight standardized to 5% lipid‐equivalent basis (Panel B). Biomagnification factors are presented on a lipid‐normalized basis (Panel C). The BAT in silico data (i.e., model predictions) are presented in the gray section of the figure panels at the far right and do not include RS. The BAT‐calculated B‐metrics (laboratory BCF, field BAF, laboratory BMF, field BMF) were derived using the partitioning data presented in the Supplemental Data Table S2‐1 and the geometric mean of the whole body biotransformation rate data. Table 1 summarizes the SoE results for different B categorization outcomes for all LoE with RS > 0. The SoE summary for the BCFs and BAFs is shown on a wet weight and wet weight standardized to 5% lipid‐equivalent basis. The SoE for BMFs and TMFs are based on lipid‐normalized metrics.

**Table 1:** Strength of evidence summary for fish, invertebrate, and in silico data using wet weight and 5% lipid‐equivalent BCFs and BAFs (L/kg‐ww) and lipid‐normalized BMFs (kg‐lw/kg‐lw) for data points with RS > 0

Strength of evidence: BCFs and BAFs (L/kg‐ww)					
Scenario	Organism type	*n* LoE^a^	nB	B	vB
Bioaccumulation					
(BCF, BAF)	Fish	13	100.0%	0.0%	0.0%
Lab, field	Invertebrates	11	90.9%	9.1%	0.0%
	All	24	95.8%	4.2%	0.0%
Biomagnification					
(BMF, TMF)	Fish	7	100.0%		0.0%
Lab, field	Invertebrates	6	100.0%		0.0%
	All	13	100.0%		0.0%
BAT in silico					
(BCF, BAF, lab and field BMF)	Fish	4	100.0%	0.0%	0.0%
All data					
(BCF, BAF, BMF, TMF)	Fish	24	100.0%	0.0%	0.0%
Lab, field, in silico	Invertebrates	17	94.1%	5.9%	0.0%
	All	41	97.6%	2.4%	0.0%
**Strength of evidence—BCFs and BAFs (L/kg‐ww) standardized to 5% lipid‐equivalent**					
Bioaccumulation					
(BCF, BAF)	Fish	11	100.0%	0.0%	0.0%
Lab, field	Invertebrates	13	61.6%	30.8%	7.6%
	All	24	79.2%	16.7%	4.2%
Biomagnification					
(BMF, TMF)	Fish	7	100.0%		0.0%
Lab, field	Invertebrates	6	100.0%		0.0%
	All	13	100.0%		0.0%
BAT in silico					
(BCF, BAF, lab and field BMF)	Fish	4	100.0%	0.0%	0.0%
All data					
(BCF, BAF, BMF, TMF)	Fish	22	100.0%	0.0%	0.0%
Lab, field, in silico	Invertebrates	19	73.7%	21.0%	5.3%
	All	41	87.8%	9.8%	2.4%

B = bioaccumulative; BAF = bioaccumulation factor; BAT = Bioaccumulation Assessment Tool; BCF = bioconcentration factor; LoE = lines of evidence; lw = lipid weight; nB = nonbioaccumulative; RS = reliability score; SoE = strength of evidence; TMF = trophic magnification factor; vB = very bioaccumulative; ww = wet weight.

^a^ Note that the number of values used for BCF and BAFs (*n* LoE) in the wet weight and 5% lipid‐equivalent SoE are different because some studies reported lipid content, and some did not.

All empirical BCFs, BAFs, BMFs, and TMFs with RS > 0 for fish indicate that PHE is “nB” following both scenarios outlined in the problem formulation stage (i.e., BCF and BAFs < 2000 L/kg and BMFs < 1, TMFs < 1). This conclusion is reached whether the outcome is determined using wet weight BCFs and BAFs or wet weight standardized to 5% lipid‐equivalent BCFs and BAFs. The measured B data are also consistent with the biotransformation rate data as inferred in the model simulation, which indicates this process is a quantitatively important elimination route. Specifically, the measured in vivo data agree with the in silico calculations (predicted BCF, BAF, lab and field BMF) for fish. For example, the predicted wet weight laboratory BCF generated by the BAT for a 5% lipid fish is 550 L/kg (range = 300–1010 L/kg considering uncertainty in biotransformation half‐life), whereas the geometric mean of the empirical BCFs with RS > 2 is 630 L/kg both on a wet weight and 5% lipid‐equivalent basis (range = 52–1760 L/kg). This supports the conclusions that 1) biotransformation is driving the reduction of the bioaccumulation potential of PHE, and 2) dietary uptake is limited, thereby preventing biomagnification.

The B categorizations for invertebrates are dependent on whether wet weight or 5% lipid‐equivalent BCFs and BAFs are used. On a wet weight standardized to 5% lipid‐equivalent basis, 2 of 6 BCFs with RS > 0 exceed B criteria for BCF ( >2000 L/kg), and 3 out of 7 of the 5% lipid‐equivalent standardized BAFs with RS > 0 exceed the B criteria for BAF ( >2000 L/kg). In both instances, the majority of LoE are below the criteria. Furthermore, all invertebrate BAFs with RS > 0 are well below the B criteria, if wet weight values without 5% lipid‐equivalent standardization are used. This difference in SoE results is simply due to the relatively low lipid contents in these organisms (0.38%–1.41%) (Takeuchi et al. 2009). All lipid‐normalized invertebrate BMFs with RS > 0 are below the BMF criterion of 1. With respect to the BCF and BAF data, the findings are consistent with observations that most fish species readily biotransform PAHs, whereas biotransformation capacity for PAHs may vary across invertebrate species (Frank et al. 1986; Landrum 1988; Meador et al. 1995). For organisms with negligible biotransformation capacity, the wet weight BCF approaches the EQP‐based value and, as already discussed, is a function of body composition (e.g., lipid and protein content), water temperature, and salinity. The majority of the 5% lipid‐equivalent invertebrate BCFs and BAFs exceeding the B criteria are for mollusks (e.g., *M. edulis, Perna viridis, Xenostrobus securis, Crassostrea gigas*) (Baussant et al. 2001; Takeuchi et al. 2009). This finding is not unexpected given the lower reported biotransformation capacity of this organism class for PAHs when compared to crustaceans and fish (Replinger et al. 2017). Further, there appear to be significant differences in biotransformation capacity between closely related species of copepods (e.g., *Calanus finmarchicus* = 260 L/kg vs *Calanus hyperboreus* =2970 L/kg; both 5% lipid‐equivalent standardized values) (Jensen et al. 2012; Agersted et al. 2018). Although some knowledge about invertebrate biotransformation enzyme systems is available (e.g., Meador et al. 1995; Snyder 2000; Sole and Livingstone 2005; Rewitz et al. 2006), many data gaps and uncertainties remain.

There was 1 field invertebrate BMF > 1 (zooplankton, BMF_L_ = 1.6) in the compilation (Moermond et al. 2007) prior to data quality evaluations. The statistical significance of this exceedance cannot be calculated from the underlying study because sample sizes (number of measured concentrations in zooplankton and diet) were not reported, only standard deviations. The zooplankton BMF_L_ was calculated using assumed dietary preferences that may not be correct. Bias in the reported concentrations may also exist due to collection or separation methods that may not accurately distinguish zooplankton from dietary sources. From the reported concentrations and standard deviations in zooplankton (4.7 ± 2.3 mg/kg lipid) and the 2 assumed prey items (20% of diet = 4.6 ± 2.3 mg/kg lipid; 80% = 2.6 ± Not Reported mg/kg lipid), the BMF_L_ and propagated error is 1.6 ± 1.4 (i.e., logically inconclusive). Because of these shortcomings and large uncertainties, this LoE was assigned an RS = 0 (Critical Fail, Not Reliable).

### Evidence integration: Fugacity ratios

**Figure 2:**
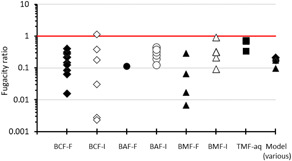
Fugacity ratios for PHE from measured and modeled B‐metrics for a range of species (fish = closed markers; invertebrates = open markers). B = bioaccumulative; BAT = Bioaccumulation Assessment Tool; BCF‐F = bioconcentration factor‐fish; BCF‐I = BCF‐invertebrate; BAF‐F = bioaccumulation factor‐fish; BAF‐I = BAF‐invertebrate; BMF‐F = biomagnification factor‐fish; BMF‐I = BMF‐invertebrate; PHE =phenanthrene; TMF‐aq = trophic magnification factor aquatic food webs. Model output (BAT in silico B assessment) includes laboratory BCF, field BAF, and lab and field BMF for fish.

Figure 2 summarizes BAT calculated fugacity ratios for the measured laboratory and field LoE for fish and invertebrates for all LoE with RS > 0 and modeled fish B‐metrics (i.e., BCF, BAF, lab and field BMF). For studies from marine environments (i.e., salinity = 35 practical salinity units [PSU]), the fugacity ratios were calculated manually to account for the “salting out” phenomenon. Except for 1 invertebrate BCF, all the empirical and modeled fugacity ratios are less than 1, and collectively the WoE of the data show that PHE does not biomagnify in food webs. The invertebrate BCF with a fugacity ratio slightly above 1 is the reported value for *M. edilus* in seawater at 7 °C (Baussant et al. 2001). The small discrepancy between the theoretical maximum fugacity ratio for a BCF and the calculated value likely reflects measurement uncertainty and indicates that the concentrations of PHE in these organisms are near equilibrium with concentrations in water.

## DISCUSSION

This critical review and application of the BAT to conduct a B assessment of PHE has highlighted many of the considerable challenges and complexities associated with regulatory decision‐making for a data‐rich substance like PHE. Recommendations to address these issues which are relevant to B assessment in general are provided first, and summary conclusions for the B assessment of PHE follow.

### General recommendations for B assessment

The OECD WoE guidance (OECD 2019) formalized into frameworks like the BAT provide opportunities for consistent and transparent data analysis and decisions, whereas applications of these frameworks will help to identify key issues that require clarification, refinement, and resolution for a scientifically defensible regulatory policy that is consistent and objective. First and foremost, only relevant and reliable quality data should be used in a WoE approach (OECD 2019), particularly when multiple LoE are available. For B assessment, it is important to ensure the units of the data (LoE) correspond to the units of the regulatory criteria. Using lipid‐normalized B‐metrics, that is, BCFs or BAFs with units of L/kg‐lw, to compare against B criteria for wet weight tissue concentration, that is, BCFs or BAFs with units of L/kg‐ww, is demonstrably incorrect. For relatively low lipid content organisms (i.e., ≤2% lipid weight on a whole body wet weight basis), lipid‐equivalent methods that account for the sorption capacity of nonlipid phases, that is, proteins, for lipid‐normalization and lipid standardization calculations should be considered to avoid introducing bias in the B metric. If invertebrate laboratory data are used in regulatory decision‐making (e.g., ECHA 2018), there is a need for standardized testing guidance and data reporting requirements for the species being considered. Furthermore, if invertebrate data and lipid‐standardization methods are to be considered, there needs to be an appropriate lipid content justifiably selected for lipid standardization (or lipid‐equivalent standardization). Despite challenges and sources of error with field B‐metrics, standard measurement and data quality guidelines for field data are still lacking. There is also a need for guidance on how to appropriately address variability in environmental conditions (i.e., temperature, pH, and salinity) that can influence certain B‐metrics for comparisons against B categorization criteria. The recommendation that measured values be standardized to general conditions in which the criteria were originally derived (Matthies et al. 2016) is both pragmatic and consistent with current guidance for 5% lipid standardization (Arnot and Gobas 2006; Arnot et al. 2008; OECD 2012; Arnot and Quinn 2015). Following this logic for B assessment, exposures in fresh water and temperatures approximating estimates of central tendency from several thousand BCF and BMF studies (Arnot and Gobas 2006; Arnot et al. 2008; Arnot and Quinn 2015) should be considered. Risk‐based assessments should then consider environmental factors that can influence absolute tissue exposures. A final general, and critically important, recommendation for B assessment is for continued scientific and regulatory consensus building to determine and clarify the underlying objective of a B categorization (i.e., priority setting vs risk management), which can inform selection of appropriate B‐metrics and criteria that support decision‐making.

### Bioaccumulation assessment of PHE

Phenanthrene has a log *K*_OW_ of 4.47 and thus only approximates the REACH screening criterion for log *K*_OW_ of 4.5. To address a general lack of scientific and regulatory consensus for B‐metrics and criteria, the present assessment used 2 general paradigms (“BCF and BAF” or “BMF and TMF”) in the problem formulation for the B assessment of PHE, following OECD WoE guidance (OECD 2019). Fortunately, in the present case the conclusions to both questions developed in the problem formulation stage (hypotheses) yielded the same conclusion; namely, that PHE is “nB.” It is emphasized that there are similar numbers of LoE for both fish and invertebrates for both hypotheses, reflecting the inclusive scope of the assessment (Table 1).

Decision‐making should be informed by addressing uncertainty in LoE. All relevant measured LoE were collected and critically evaluated for reliability. Using reliable quality data is a critical aspect of the WoE approach. As appropriate, some LoE deemed not reliable were not used in the WoE. In absence of formal regulatory guidance, the more conservative assumptions regarding temperature, salinity, and lipid standardization for invertebrate data were intentionally selected to avoid perceptions to the contrary. Importantly, the more conservative approaches do not alter the findings of the collective WoE and only marginally influence the SoE.

With respect to BCFs and BAFs on a wet weight basis, the SoE is unanimous when only fish are considered (100% nB) and remains very high when both fish and invertebrates are considered (96% nB). When BCFs and BAFs for fish and invertebrates are converted to 5% lipid‐standardized wet weight values, the SoE is lower (79% nB), but the data are still generally consistent in terms of outcome. Invertebrate BCFs (geomean with RS > 0 = 1000 L/kg ww or 1044 lipid‐equivalent basis) are higher than the fish BCFs (geomean with RS > 0 = 630 L/kg ww or lipid‐equivalent basis). These results are consistent with the observed BMF and TMF data that show biodilution across the aquatic ecosystem. Lipid‐normalized BMFs and TMFs greater than 1 indicate a higher potential for exposure because such values indicate an increase in chemical activity (or fugacity) in aquatic organisms above what would be achieved from respiratory exposure alone (i.e., biomagnification is occurring). Following this problem formulation, PHE does not present a higher exposure potential because critical interpretation of the BAF, BMF, and TMF measurements and fugacity ratio data for fish and invertebrates indicate that PHE does not biomagnify in aquatic organisms and aquatic food webs (SoE = 100% nB for these LoE). Combining both B assessment paradigms of course also results in a high SoE that PHE is “nB.” General agreement between the BAT model‐calculated B‐metrics and the measured B‐metric builds further confidence in the overall WoE assessment. In summary, there is a coherent and compelling body of evidence encompassing in vivo laboratory data and field measurements for fish and invertebrates, in vitro biotransformation rate data for fish, and by in silico simulations generated by the BAT models, resulting in a convincing conclusion that PHE is not bioaccumulative in aquatic environments.

## SUPPLEMENTAL DATA

Supplemental Data include BAT Data Evaluation Templates (DETs), property summary and estimation approaches, fish and invertebrate study details, study‐by‐study reliability scores, and additional analyses of Carlson et al. (1979) BCF data and Khairy et al. (2014) BAF data. The BAT and accompanying User Manual and Quick Start guidance can be downloaded free of charge from https://arnotresearch.com/models/.

**Section S1.** Data evaluation templates for key Bioaccumulation Assessment Tool (BAT) lines of evidence (LoE)

**Section S2.** Physical–chemical properties and application of poly‐parameter linear free energy relationships (ppLFER)

**Section S3.** 5% Lipid and 5% lipid‐equivalent standardization of bioaccumulative (B) metrics

**Section S4.** Fish bioaccumulation data

**Section S5.** Invertebrate bioaccumulation data

**Section S6.** Study‐by‐study reliability scoring

**Section S7.** Reanalysis of Carlson et al. (1979) bioconcentration factor (BCF) studies

**Section S8.** Fugacity ratio analysis of Khairy et al. (2014) bioaccumulation factors (BAFs).

## Supporting information

This article contains online‐only Supplemental Data.

Supporting information.

Supporting information.

## Data Availability

All data used in this study are provided as Supplemental Data in the form of a pdf and an Excel workbook.
