# Supplementary material for: A critical review and weight of evidence approach for assessing the bioaccumulation of phenanthrene in aquatic environments
Source: Integr Environ Assess Manag. 2021 Mar 22;17(5):911–25. doi: 10.1002/ieam.4401 (PMC8451923; doi:10.1002/ieam.4401)
Supplement: Supplementary file 1 — Supporting information. [file IEAM-17-911-s001.pdf]

SUPPORTING INFORMATION

Title:

A Critical Review and Weight of Evidence Approach for Assessing the Bioaccumulation of Phenanthrene in Aquatic Environments

Table of Contents

SUPPORTING INFORMATION 1

Section S1. Data Evaluation Templates for Key BAT Lines of Evidence (LoE) 2

Laboratory BCF 2

Laboratory BMF 3

Field BAF & BMF 4

Field TMF 4

Section S2. Phys-Chem Properties and Application of ppLFERs 5

Section S3. 5% Lipid and 5% Lipid-Equivalent Standardization of B metrics 6

Section S4. Fish Bioaccumulation Data 8

Summary of fish data entered into the BAT 9

Section S5. Invertebrate Bioaccumulation Data 13

Summary of invertebrate data 13

Invertebrate BCF Data 5% Lipid and Lipid-EQ Standardization 15

Section S6. Study by Study Reliability Scoring 22

Fish data 22

Invertebrate data 28

Section S7. Reanalysis of Carlson et al. 1979 BCF Studies 32

Section S8. Fugacity ratio analysis of Khairy et al. 2014 BAFs 38

Literature Cited 39

Section S1. Data Evaluation Templates for Key BAT Lines of Evidence (LoE)

Laboratory BCF

Table S1- 1: Lab BCF (fish and aquatic invertebrate studies) data evaluation template (DET) with scoring

#	Quality Criterion/Consideration	Maximum Score
1	BCF units clearly reported	Pass/Fail
2	BCF for parent chemical reported	Pass/Fail
3	If BCF was calculated as C_{Fish}/C_{Water}, was the steady state assumption ("within 20%) confirmed? (otherwise N/A)	Pass/Fail
4	If BCF was calculated as k_1/k_T, were the rate constants with units clearly reported? (otherwise N/A)	Pass/Fail
5	Organism concentration measured directly for chemical of interest?	Pass/Fail
6	For ionizables, was pH reported and within 0.5 log units of average? (otherwise N/A)	Pass/Fail
7	Estimated dissolved water concentration (C_{Free}) with respect to Water Solubility (S_W)	20 (Fail if $C_{Free} > 2 \cdot S_W$)
8	Water Concentration measured directly for chemical of interest?	20
9	Water Concentration within ± 20% of nominal throughout exposure?	20
10	For log K_{OW} > 6, was TOC reported and less than 2mg/L? (otherwise N/A)	20
11	Mortality/adverse effects in test/control group < 5%	20
12	Whole-body lipid content reported?	20
13	Test species reported?	20
14	Organism mass reported?	10
15	Whole-body analyzed?	10
16	For chemicals with logKow, n > 6, was growth rate reported?	10
17	Was there a control group?	10
18	What was the chemical purity?	10
19	LOQ reported?	10
20	Study conducted according to recognized international standard e.g., OECD305?	10
21	Study consistent with GLP or similar guiding principles?	5
22	Test design (flow through, semi-static, static, not reported)	5
23	Water temperature reported AND appropriate for species AND relatively constant (±2°C)	5
24	Test concentration < 1% reported acute toxicity?	5
25	For neutrals: was pH reported?	5
26	For log K_{OW} ≤ 6, was TOC reported and less than 2mg/L? (otherwise N/A)	5
27	Was dissolved oxygen reported and > 60% saturation?	5
28	Similar size of organism used throughout study?	5
29	Acclimatization for at least 14 days under test conditions?	2
30	Feeding rate reported and appropriate?	2
31	Quantity of organisms sampled per sampling event appropriate?	2
32	Water hardness is reported AND 10-250 mg/L?	2
33	Light-dark cycle reported AND 12-16 h illumination?	2
34	Critical Fail for other reason (override; quality score = 0)	Fail

Laboratory BMF

Table S1- 2: Lab BMF (fish studies) data evaluation template (DET) with scoring

#	Quality Criterion/Consideration	Maximum Score
1	BMF units clearly reported	Pass/Fail
2	BMF for parent chemical reported	Pass/Fail
3	If BMF was calculated as C_{Fish}/C_{Diet}, was the steady state assumption ("within 20%) confirmed? (otherwise N/A)	Pass/Fail
4	If BMF was calculated as $(I \cdot E_D)/k_T$, were the rate constants with units clearly reported? (otherwise N/A)	Pass/Fail
5	Fish concentration measured directly for chemical of interest?	Pass/Fail
6	Diet Concentration measured directly for chemical of interest?	20
7	Diet lipid content reported?	20
8	Whole-body fish lipid content reported?	20
9	Feeding rate reported in the range of 1-3% body weight per day?	20
10	Was growth rate reported?	20
11	Test species reported?	20
12	Fish mass reported? Yes, Partial (start and/or end) or No	10
13	Whole-body fish analyzed?	10
14	Was there a control group?	10
15	Mortality/adverse effects in test/control group < 5%	10
16	For ionizables, was pH reported and within 0.5 log units of average? (otherwise N/A)	10
17	What was the chemical purity?	10
18	LOQ reported?	10
19	Study conducted according to recognized international standard e.g., OECD305?	10
20	Study consistent with GLP or similar guiding principles?	5
21	Test design (Flow through, semi-static, static, not reported)	5
22	Water temperature reported AND appropriate for species AND relatively constant (±2°C)	5
23	Test concentration < 1% reported acute toxicity (LC50)? If LC50 not reported = "0"	5
24	For neutrals: was pH reported?	5
25	Was dissolved oxygen reported and > 60% saturation?	5
26	Similar weight or length of fish used throughout study?	5
27	Acclimatization for at least 14 days under test conditions?	2
28	Minimum of 4 fish/sampling event?	2
29	Water hardness is reported AND 10-250 mg/L? Light-dark cycle reported AND 12-16 h illumination?	2
30	BMF units clearly reported	2
31	Critical Fail for other reason (override; quality score = 0)	Fail

Field BAF & BMF

Table S1- 3: Field BAF and BMF (fish and invertebrate studies) data evaluation template (DET) with scoring

#	Quality Criterion/Consideration	Maximum Score
1	Were field blanks used in the sampling?	30
2	Were water (for BAF) and dietary (for BMF) samples used in B metric co-located and considered representative of the exposures?	30
3	Were biological and environmental (e.g., water) samples used in B metric obtained in the same year and season?	30
4	Enter a score representing confidence that steady-state is approximated (e.g., +/- ~20%) on a score of 0-30 (30 being analytical confirmation of this assumption):	30
5	For BAFs: is the water concentration based on estimates for dissolved or total (bulk) water phase?	20
6	For BMFs: is diet lipid content reported?	20
7	Was the whole-body analysed? If tissue only analysed, are tissue lipid contents reported?	20
8	Was the frequency of detects 100%? 80-100%? 50-80%? < 50%? OR Unknown	20
9	Were analytical standards used in the analysis?	15
10	Is LOQ reported?	15
11	Are sampled species names reported?	15
12	For each sampled species (i.e., fish or within a taxa / TL for lower TLs): are organism masses (or length or age) reported and similar (i.e., min. differences)?	15
13	Is environmental temperature reported?	5
14	For ionizables: was pH reported?	5
15	Are locations and dates of the samples used in B metric reported?	5
16	Was a randomized sampling design employed?	5
17	Were there an adequate number of samples?	5
18	Critical Fail for other reason (override; quality score = 0)	Fail

Field TMF

Table S1- 4: Field TMF (fish and invertebrate studies) data evaluation template (DET) with scoring

#	Quality Criterion/Consideration	Maximum Score
1	Were field blanks used in the sampling?	30
2	Were biological samples used in B metric co-located and relevant for dietary relationships?	30
3	Were biological samples used in B metric obtained in the same year and season?	30
4	Enter a score (0-30) representing confidence that steady-state is approximated (e.g., +/- ~20%) with 30 being analytical confirmation of this assumption:	30
5	Did the study including sampling from a minimum trophic level range of 2.0 (i.e., TL 2.0 - 4.0)?	30
6	Was the method used to derive trophic level provided (e.g., δ15N /δ13C stable isotope ratio data available and appropriate baseline organism used)?	30
7	Was the method used to determine the TMF provided?	30
8	Did the study design incorporate reasonable balance with respect to sample numbers of lower- versus higher-trophic-level organisms?	20
9	Were lower trophic level organisms included in sampling (e.g., non-vertebrates)?	20
10	Was the whole-body analysed? If tissue only analysed, was a correction (normalization)	20

#	Quality Criterion/Consideration	Maximum Score
	performed?	
11	Were the sample concentrations normalized appropriately?	20
12	Was the frequency of detects 100%? 80-100%? 50-80%? < 50%? OR Unknown	20
13	Analytical standards used in the analysis?	15
14	Is LOQ reported?	15
15	Are sampled species names reported?	15
16	For each sampled species (i.e., fish or within a taxa / TL for lower TLs): organism mass (or length or age) reported and similar (i.e., min. differences)?	15
17	Is environmental temperature reported?	5
18	For ionizables: was pH reported?	5
19	Are locations and dates of the samples used in B metric reported?	5
20	Was a randomized sampling design employed?	5
21	Were there an adequate number of samples?	5
22	Critical Fail for other reason (override; quality score = 0)	Fail

Section S2. Phys-Chem Properties and Application of ppLFERs

Property data compiled from the literature [1] and estimated for PHE using polyparameter linear free energy relationships (ppLFERs) are summarized in Table S2-1. Details of the ppLFER calculations are documented below. Setschenow constant estimated as per Ni and Yalkowsky (0.04 log K_{OW} + 0.114) [2].

Table S2- 1: Property and partitioning data at 25 °C selected for phenanthrene.

Property	Units	Value
Molecular weight (MW)	g/mol	178.23
Vapour Pressure (VP)	Pa	0.026
Water solubility (S_W)	mg/L	1.09
Setschenow constant		0.293
Partitioning		
log octanol-water (log K_{OW})	L/L	4.47
log air-water (log K_{AW})	L/L	-2.76
log octanol-air (log K_{OA})	L/L	7.64
Biopartitioning		
log storage lipid-water (log K_{SlW})	L/L	4.81
log membrane lipid-water (log K_{MW})	L/L	4.70
log serum albumin-water (log K_{SaW})	L/L	3.95
log structural protein-water (log K_{PW})	L/L	3.27
Temperature Dependence		
Octanol-water partitioning (ΔU_{OW})	kJ/mol	-20.5
Air-water partitioning (ΔU_{AW})	kJ/mol	53.4
Octanol-air partitioning (ΔU_{OA})	kJ/mol	-73.9
Storage lipid-water partitioning (ΔU_{SlW})	kJ/mol	-20.3

We relied on the UFZ LSER database (http://www.ufz.de/lserd) to obtain the required solute descriptors (E,S,A,B,L) and equations to estimate bio-partition coefficients at 37 °C. The LSER database contains multiple estimates of the solute descriptors for phenanthrene. For the following calculations, we selected the values published by Abraham et al. [3]. For each bio-partition coefficient, the LSER database provides two equations which differ in the solute descriptors used for the regression against the training set. The average of the two values was selected for further use in the BAT. Solute descriptors and estimated bio-partition coefficients at 37 °C are summarized in Table S2- 2.

Table S2- 2: Solute descriptors and estimated bio-partitioning coefficients at 37°C

**Solute Descriptors**	**Bio-partition Coefficient**	EQN 1	EQN 2
E 2.06	log storage lipid-water	4.58	4.76
S 1.29	log membrane-water	4.50	4.63
A 0.00	log serum albumin-water	3.78	3.84
B 0.29	log structural protein-water	3.00	3.26
V 1.45			
L 7.63			

Geisler et al. [4] examined the influence of fatty acid composition and temperature on the partitioning properties of various surrogate storage lipids. The authors reported the following ppLFER for estimating the internal energy of phase change for storage lipids (ΔU_{SlW}, kJ/mol).

$$\Delta U_{SlW} = 10.51L - 49.29S - 16.36A + 70.39B - 66.19V + 38.95$$

Using this equation, the estimated value for phenanthrene is -20.3 kJ/mol, very similar to the internal energy of phase change for octanol-water partitioning we would have applied otherwise (-20.5 kJ/mol) [5]. The ΔU_{SlW} of -20.3 kJ/mol corresponds to an increase in the storage lipid partition coefficient by a factor of approximately 1.4 (0.14 log units) from 37 to 25°C. Because of the similarity between the temperature dependence of storage lipid and octanol-water partitioning, ΔU_{SlW} was applied to adjust all other bio-partition coefficients. As sorption to storage and membrane lipids dominate for phenanthrene, any associated error related to this assumption is minimal.

Temperature corrections are conducted using the van't Hoff equation [6], shown here for K_{OW}

$$logK_{OW(T)} = logK_{OW(REF)} + \frac{\Delta U_{OW}}{R \cdot \ln(10)} \cdot (\frac{1}{T_{REF}} - \frac{1}{T_T})$$

where log $K_{OW(T)}$ is the value of the partition coefficient at the temperature of interest (T_T, K), log $K_{OW(REF)}$ is the value of the partition coefficient at the reference temperature (T_{REF}, K) and R is the gas law constant (kJ/K/mol).

Section S3. 5% Lipid and 5% Lipid-Equivalent Standardization of B metrics

The equation for 5% lipid standardization of BCFs (and BAFs) from the OECD 305 guidelines is shown below.

$$BCF5\% = BCF_{WW} \cdot \frac{0.05}{f_L}$$

where BCF5% is the wet weight BCF for a 5% lipid content fish (L/kg), BCFww is the wet weight BCF (L/kg) of a given observation and f_L is the organism lipid content for that data point.

To accomplish a “lipid-equivalent” standardization including sorption to lipids and structural protein (e.g., muscle) the following equation can be used instead.

$$BCF5\%EQ = BCF_{WW} \cdot \frac{0.05}{(f_L + \varphi f_P)}$$

where f_P is the structural protein content of the organism and φ is the proportionality between the protein and lipid-water partition coefficients (i.e., $\varphi = K_{PW} / K_{SLW}$ where K_{PW} and K_{SLW} are protein-water and storage lipid-water partition coefficients respectively).

The general implications of including or excluding protein fractions in the lipid standardization approach is documented in Table S3.1. For the purposes of this example, all organisms are assumed to be composed of storage lipids, structural proteins and water where the protein fractions were assumed to be 0.05, 0.10, 0.15 and 0.20.

From Table S3-1, the proportionality constant for proteins vs lipid for PHE is $10^{3.27}/10^{4.81}$ ~ 0.03. The lipid-equivalent content of each organism is therefore $f_L + 0.03\ f_P$, where f_L and f_P are lipid and protein contents. 5% lipid-equivalent standardization is then BCFww or BAFww · (0.05 / ($f_L + 0.03\ f_P$). Example comparisons to 5% lipid ONLY standardization is provided along with the ratio for a BCFww of 500 L/kg.

Table S3-1. Generic example - Comparison of %5 lipid-EQ and %5 lipid standardized BCFs

Lipid Content (f_L)	Protein Content (f_P)	BCFww (L/kg)	5% lipid-EQ standardized BCF	5% lipid standardized BCF	RATIO 5% / 5%EQ BCF
0.005	0.05	500	3881	5000	1.29
0.01	0.05	500	2185	2500	1.14
0.02	0.05	500	1166	1250	1.07
0.03	0.05	500	795	833	1.05
0.04	0.05	500	603	625	1.04
0.05	0.05	500	486	500	1.03
0.10	0.05	500	246	250	1.01
0.005	0.10	500	3171	5000	1.87
0.01	0.10	500	1940	2500	1.43
0.02	0.10	500	1092	1250	1.22
0.03	0.10	500	760	833	1.14
0.04	0.10	500	583	625	1.11
0.05	0.10	500	473	500	1.09
0.10	0.10	500	243	250	1.04
0.005	0.15	500	2681	5000	1.87
0.01	0.15	500	1745	2500	1.43
0.02	0.15	500	1028	1250	1.22
0.03	0.15	500	728	833	1.14
0.04	0.15	500	564	625	1.11
0.05	0.15	500	460	500	1.09
0.10	0.15	500	240	250	1.04
0.005	0.20	500	2322	5000	2.15
0.01	0.20	500	1585	2500	1.58
0.02	0.20	500	970	1250	1.29
0.03	0.20	500	699	833	1.19
0.04	0.20	500	546	625	1.14
0.05	0.20	500	448	500	1.12
0.10	0.20	500	236	250	1.06

As illustrated in Table S3-1, there can be substantial error introduced in the 5% lipid standardization for leaner organisms e.g., a factor of nearly two for the organisms with a lipid content of 0.5% and protein content of 15%. In the case of the 1% lipid content organism, the difference between the two standardization approach results in a different conclusion regarding B i.e., “nB” if 5%-lipidEQ standardized vs. “B” is 5%-lipid standardized.

Section S4. Fish Bioaccumulation Data

Ancillary data for the fish B data were sufficient such that most of the studies could be entered directly into the BAT for automatic processing and standardization. Tables Table S4- 1 and Table S4- 2 summarize the details pertaining to the mixtures assessed in the Laboratory Fish BCF and BMF studies.

Table S4- 1: Details of chemical mixtures analyzed in Laboratory Fish BCF studies

Study	Mixture	Details
Baussant et al. [9]	yes	BAL150 (Blended Arabian Light oil), PHE 1.16%
Baussant et al. [10]	yes	BAL150 (Blended Arabian Light oil), PHE 1.16%, North Sea crude oil
Carlson et al. [11]	Sometimes, details in SI Section S7. Reanalysis of Carlson et al. 1979 BCF Studies	
Cheikyula et al. [12]	yes	mix of 4 PAHs: 30ug/L PHE, 30 ug/L PYR, 30 ug/L CHR, 3 ug/L BaP, solvent acetone:dimethylsulfoxide (acetone evap'd)
Freitag et al. [13]	unclear	100 radiolabelled chemicals assessed
Jonsson et al. [14]	yes	high and low concs mixture of NAP, 2-Methylnap, 1,3,Dimethylnap, 2-Isopropylnap, PHE, 9-methylphe, 9-ethylphe, pyrene. Sum PAH 72.31 and 7.57 ug/L
Kobajashi et al. [15]	yes	* 20ng/L sumPAH nominal
Li et al. [16]	yes	PHE, ANTH, FLUOR, PYR were released to tanks by passive dosing and 3x3 factorial OC compositions/concentrations
Lo et al.[17]	yes, dietary BCF	11 incl. HCB, trans-decalin (controls), PHE, 2,6-dimithyldecane, 2,3 dimethylheptane, 1-methylphenanthrene, n-dodecane, n-nonane, 2,2,4,6,6-pentamethylheptane, 1,3,5-trimethlbenzene, 1,3,5-trimethylcyclohexane
Wang et al. [18]	yes	PHE & ANTH, passive dosing
Wang et al. [19]	unclear, likely	deuterated PHE, ANTH, FLUOR and PYR, described as "PAHs-d10"
Xia et al. [20]	yes	PHE, ANTH, FLUOR, PYR at 3 concs (10, 5, 1 ug/L)

PHE = Phenanthrene, PYR = pyrene, CHR = chrysene, BaP = benzo[a]pyrene, NAP = naphthalene, ANTH = Anthracene, FLUOR = Fluoranthene

Table S4- 2: Details of chemical mixtures analyzed in Laboratory Fish BMF studies

Study	Mixture	Details
Gobas et al [21]	yes, dietary BCF	11 incl. HCB, trans-decalin (controls), PHE, 2,6-dimithyldecane, 2,3 dimethylheptane, 1-methylphenanthrene, n-dodecane, n-nonane, 2,2,4,6,6-pentamethylheptane, 1,3,5-trimethlbenzene, 1,3,5-trimethylcyclohexane Utilizing the same study as Lo et al. [17]
Wang et al. [19]	unclear, likely	deuterated PHE, ANTH, FLUOR and PYR, described as "PAHS-d10"

Summary of fish data entered into the BAT

Note: For all lipid-equivalent (lipid-EQ) calculations, protein content was assumed to be 15% unless other data available and the proportionality for partitioning to protein versus lipid (ϕ) is $10^{3.27}$ / $10^{4.81}$ ~ 0.03 (i.e., the ratio of the two partition coefficients). See SI Section S3.

Table S4- 3: Wet weight and estimated 5% lipid-EQ standardized wet weight fish BCFs (n = 17)

#	Study	Species	% Lipid, ww	Study Temp. (°C)	BCFs entered (L/kg ww)		5% lipid-EQ Standardized BCFs (L/kg ww)	
					Steady State	Kinetic	Steady State	Kinetic
1	Baussant et al. [9]	Turbot-*Scophthalmus maximus*	3	19	309		450	
2	Baussant et al. [10]	Turbot-*Scophthalmus maximus*	3.6	20		936		1160
3	Carlson et al. [11]	Fathead minnow *Pimphales promelas*	3.8	24	5100		6024	
4	Carlson et al. [11]	Fathead minnow *Pimphales promelas*	4.3	24	3050		3222	
14	Carlson et al. [11]	Fathead minnow-*Pimphales promelas*	4.8	24	2500		2388	
15	Carlson et al. [11]	Fathead minnow-*Pimphales promelas*	4.1	24	2000		2206	
16	Carlson et al. [11]	Fathead minnow-*Pimphales promelas*	4.4	24	1900		1965	
5	Freitag et al. [13]	Golden ide-*Leuciscus idus melanotus*	NR	23	1760		-	-
6	Jonsson et al. [14]	Sheepshead minnow-*Cyprinodon variegatus*	9.7	25	1623	2229	800	1100
7	Jonsson et al. [14]	Sheepshead minnow-*Cyprinodon variegatus*	9.7	25	700	810	345	400

#	Study	Species	% Lipid, ww	Study Temp. (°C)	BCFs entered (L/kg ww)		5% lipid-EQ Standardized BCFs (L/kg ww)	
					Steady State	Kinetic	Steady State	Kinetic
8	Cheikyula et al. [12]	Red sea bream	6	15*	182		141	
9	Li et al. [16]	Zebrafish-*Danio rerio*	5	23	900	913	828	840
10	Lo et al. [17]	Rainbow trout-*Oncorhynchus mykiss*	2.8	13.6		690		1067
11	Wang et al. [18]	Zebrafish-*Danio rerio*	5.1	15*	527	584	476	527
12	Kobayashi et al. [15]	*Pseudopleuronectes yokohamae*	2.7	17.2		1040		1660
13	Xia et al. [20]	Zebrafish-*Danio rerio*	1.5	22		585		1513
17	Wang et al. [19]	Zebrafish-*Danio rerio*	3.9	23	52		60	

*No reported water temperature so 15°C is assumed as a default.

Table S4- 4: Estimated wet weight (ww) and 5% lipid-EQ standardized fish BAFs (L/kg, n = 22)

Study	Study	Species	Lipid %	Temperature (°C)	BAF ww L/kg	BAF 5% lipid-EQ
1	Burkhard et al. [22]	Lake Trout	6.2 – 11.5	15*	20	8 – 15
2	Khairy et al. [23]	*Lepomis gibbosus*	2.4	15*	2640	4660
		Fundulus diaphanus	2	15*	520	1068
		Lepomis macrochirus	3.3	15*	2410	3228
		Hybognathus regius	3.5	15*	3330	4233
		Esox americanus	1	15*	520	1814
		Fundulus diaphanus	2	15*	2800	5755
		Morone americana	4	15*	3960	4466

Study	Study	Species	Lipid %	Temperature (°C)	BAF ww L/kg	BAF 5% lipid-EQ
		Anguilla rostrata (11-12 cm)	1	15*	1700	5933
		Anguilla rostrata (28-110 cm)	6	15*	5580	4337
		Hybognathus regius	3.5	15*	15100	19198
		Morone saxatilis (18-20 cm)	1.6	15*	1920	4723
		Morone americana	4	15*	8800	9926
		Morone saxatilis (9.6-10.4 cm)	1	15*	1000	3490
		Morone saxatilis (20-33 cm)	1.65	15*	742	1781
		Hybognathus regius	3.5	15*	12600	16019
		Dorosoma cepedianum	1.1	15*	5280	17225
		Fundulus heteroclitus	1.1	15*	5830	19020
		Menidia menidia (2.2-3.6 cm)	2.8	15*	7280	11260
		Menidia menidia (7.8-9.4 cm)	2.4	15*	10300	18181
3	Takeuchi et al. [24]	*Acanthogobius flavimanus*	0.3	15*	61.2	417
4	Ke et al. [25]	Crucian Carp	NR	15*	619	-

*No reported water temperature so 15°C is assumed as a default.

NR – Not reported

Table S4- 5: Estimated wet weight (ww) and lipid normalized (lw) field BMF data for fish (n=2)

Study	Study	Species	Lipid (%)	Lipid diet (%)	Temp (°C)	BMF ww	BMF lw
1	Nfon et al. [26]	Herring	0.58		15*		0.29
2	Moermond et al. [27]	Fish	2	3.5	15*	0.0035	0.00677

*No reported water temperature so 15°C is assumed as a default

Table S4- 6: Estimated wet weight (ww) and lipid normalized (lw) laboratory BMF data for fish (n=2).

Study	Study	Species	Lipid (%)	Lipid diet (%)	Temp (°C)	BMF ww	BMF lw
1	Gobas et al. [21]	Rainbow trout-*Oncorhynchus mykiss*	2.8	15.6	13.6	0.0111	0.0658
2	Wang et al. [18]	Zebrafish-*Danio rerio*	3.9	2	23	-	0.017

Table S4-7. Summary of Petersen & Kristensen 1998 BCF data for PHE in larval fish [28]. F = freshwater species, M = marine (experiments conducted with seawater). All testing was conducted in a semi-static system with renewal of water every 24h

Species	Water Temperature (°C)	Exposure Concentration C_W (mg/L)	Lipid content (wet weight)[a]	Kinetic BCFww[b] (L/kg)	BCF 5%lipid-EQ Standardized (L/kg)
Zebrafish (F)	27	0.16	0.039	1259	1449
Cod (M)	6.5	0.12	0.027	2884	4613
Herring (M)	7.5	0.15	0.030	4169	6137
Turbot (M)	15	0.17	0.035	2239	2812

a Wet weight lipid contents estimated from dry weight values using the dry/wet ratio of 0.20 from publication
b Kinetic BCFs reported on dry and lipid weight basis in Table 3 of original publication; Kinetic BCFs reported on wet weight basis in Table 5

Consistent with theoretical expectations, the 5%lipid-EQ standardized BCFs for the various larval fish show an inverse relationship with water temperature and are larger for the three marine species (cod, herring, turbot). As noted by Petersen and Kristensen, differences in biotransformation capacity related to interspecies differences and water temperature likely influence the results as well.

Although relatively stable, all reported exposure concentrations (0.70–0.94 µM; 0.12–0.17 mg/L) are within 10% of the water solubility in freshwater at 25 °C (1.09 mg/L, Table S2-1) raising the possibility of adverse effects occurring (i.e., baseline toxicity). Indeed, the authors report that, “The larvae of all species in experiments with phenanthrene and pyrene showed slight toxic responses observed as malformation (bilaterally bent chorda) toward the end of the exposure period”. The occurrence of the adverse effects in bioaccumulation experiments is undesirable as the influence on uptake and elimination kinetics is unclear. Although not necessarily a disqualifying factor (i.e., reason to determine study a Critical Fail), the observed toxicity is problematic and reduces the overall reliability of these data.

Section S5. Invertebrate Bioaccumulation Data

As noted in the Methods section, ancillary data required to convert invertebrate BCF data to wet weight and 5% lipid standardized values are not available for all the compiled studies. The following theoretical considerations were relied upon to facilitate the interpretation and standardization of the invertebrate bioaccumulation data. Although some study details are provided below, the Reliability Scoring of these data is a separate exercise and is documented in Section S5 and in the BAT spreadsheet provided as SI.

In the absence of biotransformation and rapid growth, bioconcentration factors of neutral organic chemicals in invertebrates will approach a value reflecting equilibrium partitioning (EQP) between lipids (and other sorption matrices) and water. As a first approximation, the wet weight EQP BCF can be estimated as shown below:

$BCF = f_L \cdot K_{SlW} + f_W$ or if including proteins $BCF = f_L \cdot K_{SlW} + f_P \cdot K_{PW} + f_W$

where f_L, f_P and f_W are the lipid, protein and water contents of the organism respectively and K_{SlW} and K_{PW} the relevant partition coefficients (Table 1 and Table S2- 2). The estimated wet weight EQP BCF of phenanthrene of a 5% lipid only organism at 25 °C is 3230 L/kg. Based on the internal energy of phase change in Table 1, the estimated EQP wet weight BCF of a 5% lipid only organism at 2 °C is 6390 L/kg.

Under the same assumptions, the wet weight lipid content of the organism can be approximated as the ratio of the reported BCF to K_{SLW}, i.e.,

$$f_L = \frac{BCF}{K_{SlW}}$$

This estimate can be compared to reported values or serve as an estimate if wet weight lipid contents are not reported in each study but wet weight BCFs are or can be estimated from dry weight data.

Summary of invertebrate data

Table S5-1: Estimated wet weight and 5% lipid-EQ standardized invertebrate laboratory BCFs (*n = 11*)

**Study**	**Study**	**Species**	**Lipid (%)**	**Exposure Temp (°C)**	**BCF ww**		**BCF 5% lipid-EQ standardized**	
					**Steady State**	**Kinetic**	**Steady State**	**Kinetic**
1	Baussant et al. [9]	Blue Mussel- *Mytilus edulis*	2	7	2932		6026	
2	Landrum [29]	*Pontoporeia hoyi*	6.3	4		7540		5600
3	Landrum et al. [30]	*Diporeia spp*	6.9	4		10261		6996
4	Frank et al. [31]	*Stylodrilus heringlanus*	3.1	4		1040		1477
5	Jensen et al. [32]	*Calanus finmarchicus*	NR	2			264	265
6	Agersted et al. [33]	*Calanus hyperboreus*	NR	2				2970
7	Cailleaud et al. [34]	*Eurytemora affinis*	6	10	160		124	
8	Southworth et al. [35]	*Daphnia pulex*	2.3	25	374		890	
9	Landrum & Poore [36]	*Hexagenia limbata*	1.4	20		90		411

Study	Study	Species	Lipid (%)	Exposure Temp (°C)	BCF ww		BCF 5% lipid-EQ standardized	
					Steady State	Kinetic	Steady State	Kinetic
10	Wang et al. [19]	*Daphnia magna*	1.42	23			161	
11	Landrum & Poore [36]	*Hexagenia limbata*	1.4	10		735		2005

Table S5-2: Estimated wet weight (ww) and 5% lipid-EQ standardized invertebrate field BAFs (n = 8)

Study	Study	Species	Lipid (%)	Temp (°C)	BAF ww	BAF 5% lipid-EQ standardized
1	Khairy et al. [23]	*Callinectes sapidus*	2.3	15*	3.22x10^{4}	5.9x10^{4}
2	Takeuchi et al. [24]	*Mercenaria stimpsoni*	0.38	15*	164	1147
		Mytilopsis sallei	1.28	15*	456	1311
		Mytilus galloprovincialis	1.41	15*	608	1609
		Perna viridis	0.73	15*	537	2658
		Xenostrobus securis	0.83	15*	593	2339
		Hemigrapsus penicillatus	2.76	15*	428	657
		Crassostrea gigas	1.03	15*	581	2205

*No reported water temperature so 15°C is assumed as a default.

Table S5-3: Estimated wet weight (ww) and lipid normalized (lw) invertebrate field BMFs (n=6)

Study	Study	Species	Lipid (%)	Temp (°C)	BMF kg/kg ww	BMF kg/kg lipid
1	Nfon et al. [26]	Zooplankton	0.06	15*	-	0.21
		Mysis	0.51	15*	-	0.32
		Saduria	0.21	15*	-	0.90
2	Moermond et al. [27]	oligo	2	15*	0.03	0.09
		zoo	0.04	15*		1.64
		invert	0.7	15*	0.17	0.31

*No reported water temperature so 15°C is assumed as a default.

Note that the lipid-normalized BMF for *Saduria* is reported in the main text of Nfon et al. as 1.06. However, this BMF_L is additionally adjusted by the ratio of trophic levels (TL). The lipid-normalized concentrations reported in the Supporting Information of Nfon et al. for *Saduria* and its assumed prey are 37.1 and 40.9 ng /g lipid respectively. Unusually, the trophic level of the predator as estimated by stable nitrogen is lower than its prey resulting in the increase in BMF_L when normalized by TL ratio. As stated by Nfon et al., "estimated FCMFs for the benthic food chain are likely in error, as the delta 15N method suggested a food chain structure which was not consistent with the known dietary patterns of the species." The calculation of the BMF based on lipid-normalized concentrations is therefore preferred and is in any case consistent with guidance documents and all other BMFs included in the assessment.

Invertebrate BCF Data 5% Lipid and Lipid-EQ Standardization

Note: For all lipid-equivalent (lipid-EQ) calculations, protein content was assumed to be 15% unless other data available and the proportionality for partitioning to protein versus lipid (ϕ) is $10^{3.27}$ / $10^{4.81}$ ~ 0.03 (i.e., the ratio of the two partition coefficients). See SI Section S5 for additional examples

Baussant et al. 2001 Bioaccumulation Of Polycyclic Aromatic Compounds: 1. Bioconcentration In Two Marine Species And In Semipermeable Membrane Devices During Chronic Exposure To Dispersed Crude Oil. *Environ Toxicol. Chem*., 20(6), 1175-84 [9]

Table S5-4: Relevant BCF information from Baussant et al.

**Species**	C_W µg/L	**Mixture?**	**Type**	**BCF (L/kg)**	**Wet/Dry/Lipid weight**	**Steady-state or Kinetic BCF (k_1/k_T)?**
Mytilus edulis	< 0.1	Yes (Crude oil at 1 mg/L)	Flow Though	146600	Lipid	Assumed steady-state (8 d exposure)

Exposures conducted at water temperature of 7 °C (seawater); log K_{SlW} of phenanthrene at 7 °C is 5.04

Water concentration reported via graph only; appears to be < 0.1 µg/L

Reported wet weight lipid content = 2%; lipid-normalized BCFs reported by authors

Wet weight BCF = (0.02 * 146 600) = 2932 L/kg

5% lipid standardized BCF = 146600 * (0.05/1) = 7330 L/kg

5% lipid-EQ standardized BCF = 2932 * (0.05/(0.02 + ϕ0.15)) = 6026 L/kg

5% lipid standardized BCF is greater than 5% EQP BCF at 7 °C (5455 L/kg) but K_{SlW} is referenced to freshwater and so does not account for 'salting out'-effect on partitioning; BCF deemed plausible with respect to EQP given broader uncertainties

2) Landrum, P.F. 1988. Toxicokinetics of organic xenobiotics in the amphipod, *Pontoporeia hoyi*: Role of physiological and environmental variables. *Aquatic Toxicology* 12 (3):245-271 [29]

Table S5-5: Relevant BCF information from Landrum [29]

**Species**	C_W µg/L	**Mixture?**	**Type**	**BCF (L/kg)**	**Wet/Dry/Lipid weight**	**Steady-state or Kinetic BCF (k_1/k_T)?**
Pontoporeia hoyi	0.7–7.1	No	Flow Through	28040	Dry? (Not clearly stated)	Kinetic BCF

- Radiolabelled; biotransformation reported to be negligible based on experimental data

Exposures conducted at water temperature of 4 °C; estimated log K_{SlW} of phenanthrene at 4 °C is 5.08

Reported average monthly dry/wet ratio = 0.269

Assuming kinetic BCF is on dry weight basis, the wet weight BCF ~ 7540 L/kg

Ratio of wet weight BCF to K_{SLW} (f_L) = 0.063

Reported dry weight lipid contents vary seasonally and range from approximately 0.2–0.5;

Corresponds to lipid contents on wet weight basis of approximately 0.054-0.135, estimated f_L within this range

5% lipid standardized BCF = 7540 * (0.05/0.063) = 5990 L/kg

5% lipid-EQ standardized BCF = 7540 * (0.05/(0.063+ ϕ0.15)) = 5600 L/kg

If kinetic BCF is on a wet weight basis

Ratio of wet weight BCF to K_{SLW} (f_L) is 0.23; exceeds expected range of wet weight lipid contents (see above)

5% lipid standardized BCF = 28040 * (0.05/0.23) ~ 6095 L/kg

3) Landrum, P. F., G. R. Lotufo, D. C. Gossiaux, M. L. Gedeon, and J. H. Lee. 2003. Bioaccumulation and critical body residue of PAHs in the amphipod, *Diporeia* spp: additional evidence to support toxicity additivity for PAH mixtures. *Chemosphere* 51 (6):481-9 [30]

Table S5-6: Relevant BCF information from Landrum et al. [30]

**Species**	**C_W** µg/L	**Mixture?**	**Type**	**BCF (L/kg)**	**Wet/Dry/Lipid weight**	**Steady-state or Kinetic BCF (k_1/k_T)?**
Diporeia spp.	**57**<br>105<br>214<br>383<br>638	No	Static Renewal (Daily)	**10 261**<br>8900<br>11216<br>10080<br>5376	Wet	Steady-state & Kinetic BCFs

- Radiolabelled; biotransformation <u>assumed</u> to be negligible by authors based on previous study with same species

Exposures conducted at water temperature of 4 °C; estimated log K_{SIW} of phenanthrene at 4 °C is 5.08

<u>Lipid contents not reported</u>; lipid contents of juvenile *Diporeia* spp on a dry weight basis reported for Lake Ontario and Michigan are 18-37% [37]

Assuming dry/wet ratios of 0.2 and 0.3, the estimated wet weight lipid contents range from 3.6–7.4% and 5.4%–11% respectively, 5% lipid standardized wet weight BCFs would therefore be 1.4-fold greater to 2.2-fold smaller.

Reported $LC50_{28d}$ = 95.2 µg/L, $LR50_{28d}$ = 7.2 mmol/kg. Only Experiment 1 (water concentration = 57.1 ug/L) does NOT exceed either of these thresholds, therefore results for only this experiment are assessed.

<u>All studies</u> have external water concentrations greater than 5% of the water solubility limit and result in Critical Body Residues consistent with baseline toxicity (narcosis) (1–10 mmol/kg) [MW = 178.23 g/mol; S_W = 950 µg/L].

Table S5-7: Summary of Landrum et al. [30] studies

**C_W (µg/L)**	**Reported BCF (L/kg)**	**C_{ORG} (mg/kg)**	**C_{ORG} (mmol/kg)**
57.1	10261	586	3.3
104.6	8900	931	5.2
214.4	11216	2400	13
383	10080	3860	22
637.8	5376	3430	19

Assuming a dry weight lipid content of 27.5% and a dry/wet ratio of 0.25, the wet weight lipid content is approximately 6.9%.

The wet weight BCF for in the experiment with the lowest exposure concentration is 10261 L/kg

5% lipid standardized BCF = 10261 * (0.05/0.069) = 7435 L/kg

5% lipid-EQ standardized BCF = 10261 * (0.05/(0.069+ ϕ0.15)) = 6996 L/kg

The 5% lipid-standardized BCF estimate is approximately 1.2-fold greater than the 5% EQP BCF ($0.05 * 10^{logKslw}$ = 6010 L/kg) but considered plausible given the uncertainties in the estimation procedure

4) Frank, A. P., P. F. Landrum, and B. J. Eadie. 1986. Polycyclic aromatic hydrocarbon rates of uptake, depuration, and biotransformation by Lake Michigan *Stylodrilus heringianus*. *Chemosphere* 15 (3):317-330 [31]

Table S5-8: Relevant BCF information from Frank et al. [31]

Species	C_W µg/L	Mixture?	Type	BCF (L/kg)	Wet/Dry/Lipid weight	Steady-state or Kinetic BCF (k_1/k_T)?
Stylodrilus heringianus	<200	No	Flow Through	5222	Dry	Kinetic BCF

- Radiolabelled; biotransformation reported to be negligible based on experimental data

Exposures conducted at water temperature of 4 °C; estimated log K_{SlW} of phenanthrene at 4 °C is 5.08

Study states that, "Upon removal, animals were desiccated for later analysis" – we assume this means that all biota concentrations are reported on a dry weight basis

Lipid contents not reported; expected values based on data from Lake Michigan are 12-19% (dry weight).

Assuming dry/wet ratio = 0.2, the corresponding wet weight BCF = 1044 L/kg (not lipid standardized).

Assuming dry/wet ratio = 0.2, the expected wet weight lipid contents are 2.4–3.8%

Assuming an average wet weight lipid content of 3.1%

5% lipid standardized BCF = 1044 * (0.05/0.031) = 1685 L/kg

5% lipid-EQ standardized BCF = 1044 * (0.05/(0.031+ ϕ0.15) = 1477 L/kg

5) Jensen, L. K., J. O. Honkanen, Jæ, I. ger, and J. Carroll. 2012. Bioaccumulation of phenanthrene and benzo[a]pyrene in *Calanus finmarchicus*. *Ecotoxicology and Environmental Safety* 78:225-231 [32]

Table S5-9: Relevant BCF information from Jensen et al. [32]

Species	C_W µg/L	Mixture?	Type	BCF (L/kg)	Wet/Dry/Lipid weight	Steady-state or Kinetic BCF (k_1/k_T)?
Calanus finmarchicus	1.9	No	Static Renewal (Daily)	5281	Lipid	Steady-state and Kinetic BCF (5327 L/kg)

- Radiolabelled

Exposures conducted at water temperature of 2 °C; estimated log K_{SlW} of phenanthrene at 2 °C is 5.11

Biota concentrations expressed in units of µg / g lipid
Uptake rate constants expressed in units of ml / g lipid / h

Lipid-normalized BCF is approximately 24-fold lower than K_{SLW} of phenanthrene at 2 °C; indicates that rapid biotransformation of parent compound is occurring (i.e., concentration in organism is well below equilibrium).

5% lipid standardized steady-state BCF = 5281 * (0.05/1) = 264 L/kg
5% lipid standardized kinetic BCF = 5327 * (0.05/1) = 266 L/kg

Average 5% lipid standardized BCF = 265 L/kg
Lipid-EQ standardization not conducted because no wet weight lipid content provided

6) Agersted, M. D., E. F. Møller, and K. Gustavson. 2018. Bioaccumulation of oil compounds in the high-Arctic copepod *Calanus hyperboreus*. *Aquatic Toxicology* 195:8-14 [33]

Table S5-10: Relevant BCF information from Agersted et al. [33]

**Species**	**C_W** µg/L	**Mixture?**	**Type**	**BCF (L/kg)**	**Wet/Dry/Lipid weight**	**Steady-state or Kinetic BCF (k_1/k_T)?**
Calanus hyperboreus (3 life stages)	9.5 (±4.1)	No	Static	48360 – 71077<br><br>40330 – 59339	Lipid (estimated)	Kinetic BCFs<br><br>Concentration ratios at 89h

- Radiolabelled

Exposures conducted at water temperature of 2 °C; estimated log K_{SlW} of phenanthrene at 2 °C is 5.11

NOTE: Measured concentration of phenanthrene declined 36% over 89h exposure period; substantial variability during exposure phase as indicated by standard deviation

Water not renewed during depuration phase (static) and had reported phenanthrene concentrations of 1.7±1.3 µg/L; elimination rate constants determined during depuration phase are not valid

Kinetic BCFs based on uptake and elimination rate constants estimated <u>during exposure</u> phase

Body composition estimated; not measured

Dry weight was estimated from a length:DW regression in Hirche and Mumm (1992), including all stages of C. hyperboreus.

Lipid weight was estimated by using an average value of 60.5% lipid:DW ratio for the three stages.

Estimated lipid weight of the individuals (g lipid ind−1) was used to calculate specific tissue concentrations of dodecane and phenanthrene (µg (g lipid−1)).

Lipid-normalized BCFs are within a factor of 2-3 of K_{SLW} of phenanthrene at 2 °C; suggests limited biotransformation is occurring

5% lipid standardized BCF = 48360 * (0.05/1) = 2420 L/kg
5% lipid standardized BCF = 71077 * (0.05/1) = 3555 L/kg

Average 5% lipid standardized BCF = 2970 L/kg
Lipid-EQ standardization not conducted because no wet weight lipid content provided

7) Cailleaud, K., et al., 2009. Uptake and elimination of hydrophobic organic contaminants in estuarine copepods: an experimental study. *Environmental toxicology and chemistry.* 28(2): 239-246 [34]

Table S5-11: Relevant BCF information from Cailleaud et al. [34]

**Species**	**C_W** ng/L	**Mixture?**	**Type**	**BCF (L/kg)**	**Wet/Dry/Lipid weight**	**Steady-state or Kinetic BCF (k_1/k_T)?**
Eurytemora affinis	76 C_{NOM} = 59	Yes (6 PAHs)	Flow Through	530	Dry	Not confirmed (Sampled at 86h only)

Exposures conducted at water temperature of 10 °C; log K_{SLW} of phenanthrene at 10 °C is 5.00

NOTE: Exposure concentrations not confirmed during uptake period; only measured during pre-exposure (saturation of water tanks).

Mixture (phenanthrene, pyrene, chrysene, benzo(b)fluoranthene + benzo(k)fluoranthene, benzo(a)pyrene) but all PAHs appear to be below water solubility

<u>Lipid contents not reported</u>; no useful estimates found in the literature

Assuming dry/wet ratios of 0.2 and 0.3, the corresponding wet weight BCFs are 110 and 160 L/kg respectively; suggests rapid biotransformation of phenanthrene in this organism

The organism lipid content can be estimated by multiplying the lipid/dw = 0.2 and dw/ww = 0.3 ratios to get 0.06, or 6% lipid.

Assumed 5% lipid standardized BCF = 160 L/kg * (0.05/0.06) = 133 L/kg

Assumed 5% lipid-EQ standardized BCF = 160 L/kg * (0.05/(0.06+ ϕ0.15)) = 124 L/kg

8) Southworth, G., J. Beauchamp, and P. Schmieder, 1978. Bioaccumulation potential of polycyclic aromatic hydrocarbons in *Daphnia pulex*. *Water Research*. 12(11): 973-977 [35]

Table S5-12: Relevant BCF information from Southworth et al. [35]

**Species**	**C_W** µg/L	**Mixture?**	**Type**	**BCF (L/kg)**	**Wet/Dry/Lipid weight**	**Steady-state or Kinetic BCF (k_1/k_T)?**
Daphnia pulex	30	No	Static	325	Wet	Assumed steady-state but kinetic BCF also reported (374 L/kg)

Exposures conducted at water temperature of 25 °C; log K_{SlW} of phenanthrene at 25 °C is 4.81

200 animals per 6 L for bioaccumulation curve experiments; 25 animals in 500 ml for concentration factor experiments.

<u>Lipid contents not measured</u> but inferred to be 1-3.5% based on the ratio of BCF to K_{OW}; these estimates are broadly consistent with expectations according to Southworth et al.

5% lipid standardized BCF = 325 * (0.05/0.01) = 1625 L/kg
5% lipid standardized BCF = 325 * (0.05/0.035) = 465 L/kg
5% lipid standardized BCF = 374 * (0.05/0.01) = 1870 L/kg
5% lipid standardized BCF = 374 * (0.05/0.035) = 535 L/kg

Average 5% lipid standardized BCF = 1200 L/kg

5% lipid-EQ standardized BCF = 325 * (0.05/(0.01+ ϕ0.15)) = 1132 L/kg
5% lipid-EQ standardized BCF = 325 * (0.05/0.035+ ϕ0.15)) = 413 L/kg
5% lipid-EQ standardized BCF = 374 * (0.05/(0.01+ ϕ0.15)) = 1305 L/kg
5% lipid-EQ standardized BCF = 374 * (0.05/(0.035+ ϕ0.15)) = 475 L/kg

Average 5% lipid-EQ standardized BCF = 890 L/kg

9,11) Landrum, P.F. and Poore, R. 1988. Toxicokinetics Of Selected Xenobiotics In *Hexagenia limbata*. *J. Great Lakes Res*.. 14(4):427-437 [36]

Table S5-13: Relevant BCF information from Landrum and Poore [36]

Species	C_W µg/L	Mixture?	Type	BCF (L/kg)	Wet/Dry/Lipid weight	Steady-state or Kinetic BCF (k_1/k_T)?
Hexagenia limbata	NR	No	Flow Through	493 at 20°C	Dry?	Kinetic BCF (Based on average uptake and elimination rate constants; see below)
Hexagenia limbata	NR	No	Flow Through	4094 at 10°C	Dry?	Kinetic BCF (Based on average uptake and elimination rate constants; see below)

- Radiolabelled; biotransformation reported to be negligible based on experimental data

Exposures conducted at water temperature of 10–20°C to correspond with ambient water temperature at time of collection of specimens (May to November) from Lake St. Clair; log K_{SLW}s of phenanthrene at 10 °C and 20 °C are 5.00 and 4.87 respectively

Dry weight lipid contents reported to range from 3.3–15%; Reported average dry/wet ratio = 0.18

Reported uptake rate constants ranged from 11.9 ml/g/h (20 °C) to 131 ml/g/h (10 °C); elimination rate constants varied from 0.026 per h (10 °C) to 0.065 per h (15 °C); average values used to calculate kinetic BCF are 52.5 ml/g/h and 0.032 per h

Kinetic BCFs calculated from ratio of uptake and elimination rate constants ranged from 493 L/kg (20 °C; dry weight lipid content = 3.7%) to 4094 L/kg (10 °C; dry weight lipid content = 7.8%)

Unclear whether uptake rate constant and BCFs are reported on a dry or wet weight basis

Lipid content implied by ratio of BCF and K_{SIW} at 10°C = 4094/100000 = 4.1%

Reported dry weight lipid content = 7.8%; wet weight lipid content = 7.8*0.18 = 1.4%

Implausible for wet weight BCF to be 4094 L/kg based on lipid content of 1.4%. EQP BCF = 1400 L/kg; suggests dry weight basis

Assuming kinetic BCFs are based on dry weight

the corresponding wet weight BCF at 20 °C = 493 *0.18 = 90 L/kg (wet weight lipid content = 0.66%)

the corresponding wet weight BCF at 10 °C = 4094 *0.18 = 735 L/kg (wet weight lipid content = 1.4%)

5% lipid standardized BCF at 20 °C = 90 *(0.05/0.0066) = 680 L/kg
5% lipid standardized BCF at 10 °C = 735 *(0.05/0.014) = 2625 L/kg

5% lipid-EQ standardized BCF at 20 °C = 90 *(0.05/(0.0066+ ϕ0.15)) = 411 L/kg
5% lipid-EQ standardized BCF at 10 °C = 735 *(0.05/(0.014+ ϕ0.15)) = 2005 L/kg

10) Wang et al. 2019. Dietary Uptake Patterns Affect Bioaccumulation and Biomagnification of Hydrophobic Organic Compounds in *Fish. Environ. Sci. Techno*l. 53, 4274–4284 [19]

Table S5-14: Relevant BCF information from Wang et al. [19]

**Species**	**C_W** ng/L	**Mixture?**	**Type**	**BCF (L/kg)**	**Wet/Dry/Lipid weight**	**Steady-state or Kinetic BCF (k_1/k_T)?**
Daphnia magna	140	No	Passive dosing	4265	Lipid	Assumed steady-state (24h exposure)

Exposures conducted at water temperature of 23 °C using a passive dosing system to maintain nominal concentration

Reported dry weight lipid content = 20.5%; reported wet weight lipid content = 1.42%

Lipid normalized BCF is approximately 15-fold lower than K_{SIW}; implies rapid biotransformation

Wet weight BCF = 4265 * 0.0142 = 60 L/kg

5% lipid standardized BCF = 4265 * (0.05/1) = 213 L/kg

5% lipid-EQ standardized BCF = 60 * (0.05/(0.0142 + ϕ0.15)) = 161 L/kg

Section S6. Study by Study Reliability Scoring

Fish data

Table S6- 1: Fish summary table identifying the selected value to be included (or excluded) in the weight of evidence from each study, the study reliability and identified limitations and/or critical failures for each study. Studies with a Reliability Scores (RS) of "0" are not included in the weight of evidence. An average reliability score and LoE value are calculated for each type of study and summarized for each section (BCF, BAF, BMF, TMF). SS = steady-state, K = kinetic, L = Lipid normalized, 5% = 5% lipid-EQ standardized (BCFs). n = # of observations (LoE).

BAT worksheet	B-metric	Organism	SS/K, L/5%	Selected Value	Reliability Score (RS)	Identified study limitations *critical fails in BOLD
**BCF**						
In Silico						
	this study	Generic Lab small fish	SS, 5%	550	b	BAT Model prediction
Lab						
Lab Fish BCF 1	Baussant et al. [9]	Turbot-*Scophthalmus maximus*	SS,5%	450	2.41	Mortality/adverse effects %, fish lipid and size, control group, chemical purity, TOC, and water hardness not reported; does not meet OECD guidelines.
Lab Fish BCF 2	Baussant et al. [10]	Turbot-*Scophthalmus maximus*	K, 5%	1160	3.07	Mortality/adverse effects %, control group, chemical purity, LOQ, toxicity, pH, TOC, DO, feeding rate and water hardness not reported; does not meet OECD guidelines
Lab Fish BCF 3,4,14,15,16	Carlson et al. [11]	Fathead minnow	SS, 5%<br>SS, 5%<br>SS, 5%<br>SS, 5%<br>SS, 5%	6024<br>3222<br>2388<br>2206<br>1965	**0**	**Lab Fish BCF 3: Steady State not achieved; water concentration not stable throughout expr: Reported water concentrations = 2.0 - 3.3 µg/L**<br>**Lab Fish BCF 4: as above, concs = 1.1 – 3.1 µg/L**<br>**Lab Fish BCF 14: Mixture of phenanthrene & B-napthoflavone; evidence of induction; water**

BAT worksheet	B-metric	Organism	SS/K, L/5%	Selected Value	Reliability Score (RS)	Identified study limitations<br>*critical fails in BOLD
						**concentrations varied substantially, see SI Section S7**<br>**Lab Fish BCF 15:Mixture of phenanthrene & 9-chlorophenanthrene; evidence of induction; water concentrations substantially, see SI Section S6**<br>**Lab Fish BCF 16:Mixture of phenanthrene + 4 other PAHs; evidence of induction; concentration varied, see SI Section S6**
Lab Fish BCF 5	Freitag et al. [13]	Golden ide-*Leuciscus idus melanotus*	SS	1760	2.13	Mortality/adverse effects %, portion analyzed, lipid content, control group, chemical purity, LOQ, test design, toxicity, water hardness not reported/not met; does not meet OECD or GLP standards
Lab Fish BCF 6, 7	Jonsson et al. [14]	Sheepshead minnow	SS, 5%<br>SS, 5%	800<br>345	3.59	Control group, chemical purity, LOQ, toxicity, DO, feeding rate or water hardness not reported/not met
Lab Fish BCF 8	Cheikyula et al. [12]	Red sea bream	SS, 5%	141	**0**	**Mixture (4 PAHs) in seawater with 2 compounds (chrysene, Benzo(a)pyrene) ~ >= freshwater solubility**
Lab Fish BCF 9	Li et al. [16]	Zebrafish-*Danio rerio*	K, 5%	840	4.35	Fish mass, chemical purity, toxicity, TOC not reported
Lab Fish BCF 10	Lo et al. [17]	Rainbow trout-*Oncorhynchus mykiss*	K, 5%	1067	3.26	Water concentration not reported?<br>Chemical purity, toxicity, TOC not reported/not met
Lab Fish BCF 11	Wang et al. [18]	Zebrafish-*Danio rerio*	K, 5%	527	4.09	LOQ, toxicity, pH, TOC, DO, feeding rate not reported/not met; acclimatization < 14 days.
Lab Fish BCF 12	Kobayashi et al. [15]	*Pseudopleuronectes yokohamae*	K, 5%	1660	3.80	Chemical purity, LOQ, toxicity, acclimatization period, feeding rate, water hardness and light-dark cycle not reported/not met; > 4 fish/sampling period; does not meet OECD guidelines

BAT worksheet	B-metric	Organism	SS/K, L/5%	Selected Value	Reliability Score (RS)	Identified study limitations<br>*critical fails in BOLD
Lab Fish BCF 13	Xia et al. [20]	Zebrafish-*Danio rerio*	K, 5%	1513	4.46	Toxicity, pH, TOC and acclimatization period not reported/not met
Lab Fish BCF 17	Wang et al. [19]	Zebrafish-*Danio rerio*	SS,5%	60	4.24	Fish mass, chemical purity, toxicity not reported/not met; does not meet OECD guidelines
**Summary**	**Fish BCF**	**n (LoE)**		**Average**	**Average**	
	In Silico<br>RS > 0<br>RS = 0<br>All LoE	1<br>11<br>6<br>18		550<br>925<br>2660<br>1535	b<br>3.54<br>0<br>2.21	LabBCFs with RS > 0 are lower than those with critical issues. The BAT in silico labBCF (550 L/kg) is lower than the average experimental BCF but still in reasonable agreement; all BCFs with RS > 0 indicate PHE is nB
	**BAF**					
	In Silico					
	this study	Generic field low TL fish		782	[b]	BAT Model prediction
	Field					
BAF BMF Field 1	Burkhard et al. [22]		SS, 5%	15	**0**	**5 year gap between collection of fish tissue (1991) and water samples (1986); ambiguous reporting of lipid content of tissue, fish concentrations close to LOD**<br><br>Mixed season and location; use of field blanks, analytical standards, LOQ, temperature not reported;,
BAF BMF Field 2,3,4	Khairy et al. [23]	*Lepomis gibbosus*<br>*Fundulus diaphanus*<br>*Lepomis macrochirus*<br>*Hybognathus regius*<br>*Esox americanus*<br>*Fundulus diaphanus*<br>*Morone americana*	SS, 5%	4660<br>1069<br>3228<br>4234<br>1815<br>5755<br>4467	**0**	**Very high BAF values reported here are inconsistent with the lack of biomagnification by the TMF of the same study; body of evidence shows that respiratory uptake is unlikely to be significant**

BAT worksheet	B-metric	Organism	SS/K, L/5%	Selected Value	Reliability Score (RS)	Identified study limitations *critical fails in BOLD
		Anguilla rostrata (11-12 cm)<br>*Anguilla rostrata* (28-110 cm)<br>*Hybognathus regius*<br>*Morone saxatilis* (18-20 cm)<br>*Morone americana*<br>*Morone saxatilis* (9.6-10.4 cm)<br>*Morone saxatilis* (20-33 cm)<br>*Hybognathus regius*<br>*Dorosoma cepedianum*<br>*Fundulus heteroclitus*<br>*Menidia menidia* (2.2-3.6 cm)<br>*Menidia menidia* (7.8-9.4 cm)		5933<br>4337<br>19198<br>4723<br>9926<br>3490<br>1781<br>16020<br>17226<br>19020<br>11260<br>18181		
BAF BMF Field 5	Takeuchi et al. [24]	*Acanthogobius flavimanus*	SS, 5%	418	3.04	Use of field blanks in sampling, LOQ, temperature not reported
BAF BMF Field 8	Ke et al. [25]		SS	619	3.75	Diet lipid, if whole body or tissues analyzed, sampling design and number not reported
**Summary**	**Fish BAF**	**n (LoE)**		**Average**	**Average**	
	In Silico<br>RS > 0<br>RS = 0<br>All LoE	1<br>2<br>20<br>23		782<br>520<br>7815<br>7155	b<br>3.40<br>0<br>0.31	3 of 23 LoE have RS > 0<br>BAFs with RS > 0 are substantially lower than the failed observations; BAT in silico estimates are closer to the range of reliable estimates. BAFs with RS > 0 observations indicate that PHE is nB
**BMF**				-		
	In Silico-this study					
		Generic field low TL<br>Generic small lab fish		0.174<br>0.0952	b	BAT Model Predictions
	Field					
BAF BMF Field 6	Nfon et al. [26]	herring	SS,L	0.29	1.52	LOQ, organism mass, length or age not reported or dissimilar; pH and temperature not reported; randomized sampling design not reported/not met

BAT worksheet	B-metric	Organism	SS/K, L/5%	Selected Value	Reliability Score (RS)	Identified study limitations *critical fails in BOLD
BAF BMF Field 7	Moermond et al. [27]	"fish"	SS,L	0.00677	2.41	Diet lipids not reported but could be calculated; frequency of detects, LOQ not reported; randomized sampling design not reported/not met
	Lab					
Lab BMF 2	Gobas et al. [21]	Rainbow trout-*Oncorhynchus mykiss*	K,L	0.0658	4.38	LOQ, toxicity, pH and water hardness not reported/not met
Lab BMF 3	Wang et al. [19]	Zebrafish-*Danio rerio*	K,L	0.017	4.25	Feeding rate, chemical purity, toxicity not reported/not met
**Summary**	**Fish BMF**	**n (LoE)**		**Average**	**Average**	
	In Silico<br>RS > 0<br>RS = 0<br>All LoE	2<br>4<br>0<br>6		0.135<br>0.0949<br>0<br>0.108	b<br>3.14<br>0<br>3.14	All entered BMF studies have RS > 0 and show no biomagnification (PHE is nB)
**TMF**						
	Field					
Field TMF 1	Khairy et al. [23]			0.34	3.85	Only 1 non-vertebrate included in testing; temperature, pH and exact dates of samples not reported
Field TMF 3	Nfon et al. [26]			0.74	2.88	Frequency of detects, LOQ, mass, length or age, temperature, pH not reported; sample not randomized.
Field TMF 4	Wan et al. [38]			0.43[c*]	3.59	**p-value of 0.182 for slope not significant i.e. not different from 1 indicating result cannot be B or vB. Trophic dilution also cannot be concluded. This value is not included in the average TMF, but the reliability score is included in the average reliability of the designation.**

BAT worksheet	B-metric	Organism	SS/K, L/5%	Selected Value	Reliability Score (RS)	Identified study limitations<br>*critical fails in BOLD
						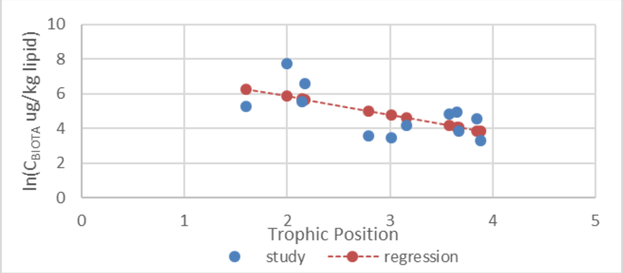
Field TMF 5	Wang et al. [39]			1.27[c*]	3.40	**p-value of 0.317 for slope not significant (i.e. not different from 1) indicating that this study cannot be concluded to be B or vB despite TMF > 1. This value is not included in the average TMF, but the reliability score is included in the average reliability of the designation.**<br>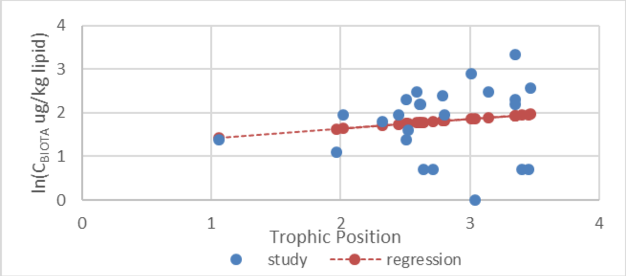
Field TMF 6	Qadeer et al. [40]			0.68	4.62	Frequency of detects not reported; randomized study design not employed
**Summary**	**Fish TMF**	**n (LoE)**		**Average**	**Average**	
	In Silico<br>RS > 0<br>RS = 0<br>All LoE	0<br>5<br>0<br>5		-<br>0.587<br>0<br>0.587	b<br>3.67<br>0<br>3.67	All entered BMF studies have RS > 0 and show no biomagnification (PHE is nB)

[a]BAT *In silico* ranges reflect the theoretical minimum and maximum values considering uncertainty in biotransformation estimates [b]BAT calculated B-metrics are not assigned a reliability score
[c]This value is not included in the average TMF, but the reliability score is included in the average reliability of the designation.
LOQ – Limits of Quantification; TOC – Total Organic Carbon in water; DO – Dissolved Oxygen content; OECD – Organization for Economic Co-operation and Development; GLP – Good Laboratory Practices.

Invertebrate data

Table S6- 2: Invertebrate summary table including wet weight and lipid standardized or normalized results, steady state and kinetic estimates, no growth correction. SS = steady-state, K = kinetic, L = 5% lipid-EQ standardized (BCFs, BAFs). n = # of observations (LoE). Most Selected values for invertebrate BCFs were 5% lipid-EQ standardized manually as documented in SI Section S4.

**BAT worksheet**	**B-metric**	**Organism**	**SS/K, L? (n)**	**Selected Value**	**Reliability Score (RS)**	**Identified study limitations *critical fails in BOLD**
**BCF**						
	Lab					
Lab Invert BCF 1	*Baussant et al. [9]*	Blue Mussel-*Mytilus edulis*	SS,5%	6026	3.44	LOQ, toxicity, pH, TOC, DO and water hardness not reported/not met.
Lab Invert BCF 2	*Landrum, [29]*	*Pontoporeia hoyi*	K, 5%	5600	**0**	**Kinetic BCF; k1 and kT were not reported clearly (units unclear).**
Lab Invert BCF 3	*Landrum et al. [30]*	*Diporeia spp*	K, 5%	6996	**0**	**Could not determine whether organism concentration not measured directly. Water concentration not measured directly. Very little study information**
Lab Invert BCF 4	*Frank et al. [31]*	*Stylodrilus heringlanus*	K, 5%	1477	2.67	Water concentration varied or nominal not reported; mortality, lipid content, mass, LOQ, TOC, DO and feeding rate not reported/not met.
Lab Invert BCF 5	*Jensen et al. [32]*	*Calanus finmarchicus*	SS, 5%	264	2.89	Mortality, lipid content, mass, LOQ, TOC, DO and feeding rate not reported/not met.
Lab Invert BCF 6	Agersted et al. [33]	*Calanus hyperboreus*	SS, 5%	2970	3.2	Organism mass, chemical purity, LOQ, TOC, DO, feeding rate and light-dark cycle not reported or not met.
Lab Invert BCF 7	Cailleaud et al. [34]	*Eurytemora affinis*	SS	124	**0**	**Mixture - although all components appear to be < individual water solubilities, enzyme induction due to exposure scenario is a possibility. Dry wt concentrations reported; Assumed dry/wet = 0.3 and lipid/dry =0.2**

BAT worksheet	B-metric	Organism	SS/K, L? (n)	Selected Value	Reliability Score (RS)	Identified study limitations *critical fails in BOLD
Lab Invert BCF 8	Southworth et al. [35]	*Daphnia pulex*	SS, 5%	890	2.54	Mortality, lipid content, mass, toxicity, pH, TOC, DO and feeding rate not reported or not met.
Lab Invert BCF 9, 11	Landrum & Poore [36]	*Hexagenia limbata*	K, 5%<br>K, 5%	411<br>2005	**0**	**Kinetic BCF; k1 and kT were not reported clearly. Organism concentration not measured directly.**
Lab Invert BCF 10	Wang et al. [19]	*Daphnia magna*	SS, 5%	161	4.24	Chemical purity, toxicity, DO not reported
**Summary**	**Invertebrate BCF**	**n (LoE)**		**Average**	**Average**	
	In Silico<br>RS > 0<br>RS = 0<br>All LoE	0<br>6<br>5<br>11		-<br>1965<br>3030<br>2450	b<br>3.16<br>0<br>1.73	Lab BCFs with RS > 0 are lower on average than those with critical issues;<br><br>One lab BCF with RS > 0 indicate that PHE is B; One lab BCF with RS > 0 indicates that PHE is vB; 4 lab BCFs with RS > 0 indicate that PHE is nB
**BAF**						
	Field			-		
BAF BMF Field 3	Khairy et al. [23]	*Callinectes sapidus*	SS, 5%	5.9×10^4	**0**	**Very high BAF values reported here are inconsistent with the lack of biomagnification by the TMF of the same study; Given partitioning properties of PHE, body of evidence suggests that dietary uptake is unlikely to be significant**

BAT worksheet	B-metric	Organism	SS/K, L? (n)	Selected Value	Reliability Score (RS)	Identified study limitations<br>*critical fails in BOLD
BAF BMF Field 5	Takeuchi et al. [24]	*Mercenaria stimpsoni*<br>*Mytilopsis sallei*<br>*Mytilus galloprovincialis*<br>*Perna viridis*<br>*Xenostrobus securis*<br>*Hemigrapsus penicillatus*<br>*Crassostrea gigas*	SS, 5%<br>SS, 5%<br>SS, 5%<br>SS, 5%<br>SS, 5%<br>SS, 5%<br>SS, 5%	1147<br>1311<br>1610<br>2658<br>2340<br>657<br>2205	3.04	Use of field blanks in sampling, LOQ, temperature not reported
**Summary**	**Invertebrate BAF**	**n (LoE)**		**Average**	**Average**	
	In Silico<br>RS > 0<br>RS = 0<br>All LoE	0<br>7<br>1<br>8		-<br>1704<br>$5.9x10^{4}$<br>8866	b<br>3.04<br>0<br>2.66	1 study with RS > 0; 7 reported values with an average 5% lipid-standardized BAF of 2420 L/kg ww; see SI Section S8
**BMF**						
	Field					
BAF BMF Field 6	Nfon et al. [26]	Zooplankton<br>*Mysis*<br>*Saduria*	SS, L<br>SS, L<br>SS, L	0.21<br>0.32<br>0.90	1.52<br>1.52<br>1.52	LOQ, organism mass, length or age not reported or dissimilar; pH and temperature not reported; randomized sampling design not reported/not met
BAF BMF Field 7	Moermond et al. [27]	"Oligotroph"<br>"Zooplankton"<br>"Invertebrate"	SS, L<br>SS, L<br>SS, L	0.0927<br>**1.64**<br>0.305	2.41<br>**0**<br>2.41	Diet lipids not reported but could be calculated; frequency of detects, LOQ not reported; randomized sampling design not reported/not met<br><br>**Moermond et al. "Zooplankton" BMF assigned Critical Fail for reasons discussed in main text (e.g., SD of BMF = ±1.4**)
**Summary**	**Invertebrate BMF**	**n (LoE)**		**Average**	**Average**	
	In Silico<br>RS > 0	0<br>6		-<br>0.605	-<br>1.97	Two reliable studies, each with 3 reported BMFs with an average of 0.605

BAT worksheet	B-metric	Organism	SS/K, L? (n)	Selected Value	Reliability Score (RS)	Identified study limitations *critical fails in BOLD
	RS = 0<br>All LoE	0<br>6		-<br>0.605	-<br>1.97	One field BMF with RS = 0 is greater than 1; issues with these studies are discussed in main text
**TMF**						
	Field					
Field TMF 2	Takeuchi et al. [24]			0.73	3.21	No method for determining TL provided; frequency of detects, temperature not reported.<br>Not determinable if randomized sampling was employed
**Summary**	**Invertebrate TMF**	**n (LoE)**		**Average**	**Average**	
	In Silico<br>RS > 0<br>RS = 0<br>All LoE	0<br>1<br>0<br>1		-<br>0.73<br>-<br>0.73	-<br>3.21<br>-<br>3.21	One reliable study with a reported TMF of 0.73.

Section S7. Reanalysis of Carlson et al. 1979 BCF Studies

Carlson et al. [11] exposed 5–6 week-old fathead minnows (*Pimephales promelas*) to various PAHs for 28 days in a flow-through system at a temperature of 24 °C. Of the five experiments including PHE, two experiments were conducted with PHE only and three as mixtures including PHE as a constituent. Concentrations in water and fish were measured directly (i.e., not radiolabelled). Mean BCFs were calculated by the authors as ratios and standard errors were estimated by accounting for variability in both observed tissue and water measurements. There was substantial variability in the reported water concentrations (~1.5–3-fold) over the 28 day exposure period in all tests (Figure S7-1) particularly for Exp#2Tank#1. OECD 305 test guideline provides test validation criteria including water concentrations of test substances are maintained +/- 20% of the average during the exposure phase (OECD 2018). Examination of Figure S7-1 shows that none of the five tests met this stringent criterion. However, failure to achieve this criterion does not automatically warrant a critical "FAIL" in the BAT, it simply deducts 20 points from the overall Reliability Scoring of the study (Table S1- 1). The range of water concentrations and Day 28 BCFs are summarized in Table S7-1 as originally reported in Carlson et al. [8].

Table S7-1: Reported phenanthrene water concentrations (µg/L) and phenanthrene BCFs at Day 28 in studies selected from Carlson et al. [11] for BAT application

EXP #	TANK #	TRIAL #	Compounds Present	C_W Mean ± Stnd. Dev. (µg/L)	C_W Range CV (%)	C_W Range (µg/L)	PHE BCF at Day 28 L/kg
2	1	1	Phenanthrene<br>*β-napthoflavone*	2.63±0.83	32	1.50 – 4.10<br>N = 8	2500±1300
2	2	1	Phenanthrene	2.55±0.44	17	2.01 – 3.27<br>N = 7	5100 ±1600
3	1	1<br>2	Phenanthrene<br>*9-chlorophenanthrene*	2.53±0.44	17	1.91– 3.26<br>N= 13	2000±600<br>2800±750
3	2	1<br>2	Phenanthrene	2.34±0.54	23	1.14 – 3.13<br>N = 12	3100±1000<br>3000±1000
3	3	1<br>2	Phenanthrene<br>*Dibenzofuran*<br>*Fluorene*<br>*1-methylphenanthrene*<br>*Fluoranthene*<br>*Pyrene*	2.20±0.25	11	1.66 – 2.49<br>N = 11	1900±500<br>2200±400[b]

a See Figure 12 of Carlson et al. 1979

b BCF SD < 20% of the mean

CV = coefficient of variation calculated and mean divided by standard deviation x 100

N = number samples analysed for phenathrene in water during the uptake period

The BCF data from the five Carlson et al. [11] experiments were entered in the BAT and assessed for reliability using the BCF DETs. These data are deemed of poor quality ("Critical Fail") because they do not fulfill Criterion #3 of the BAT Data Evaluation Template (DET) which states, "If BCF was calculated as Cfish/Cwater, was the steady-state assumption (+/-20%) confirmed?". Figure 12 of Carlson et al. [11] show that this criterion is not fulfilled which is a further validity criterion that needs to be met based on OECD 305 test guideline on BCF testing and reporting [41].

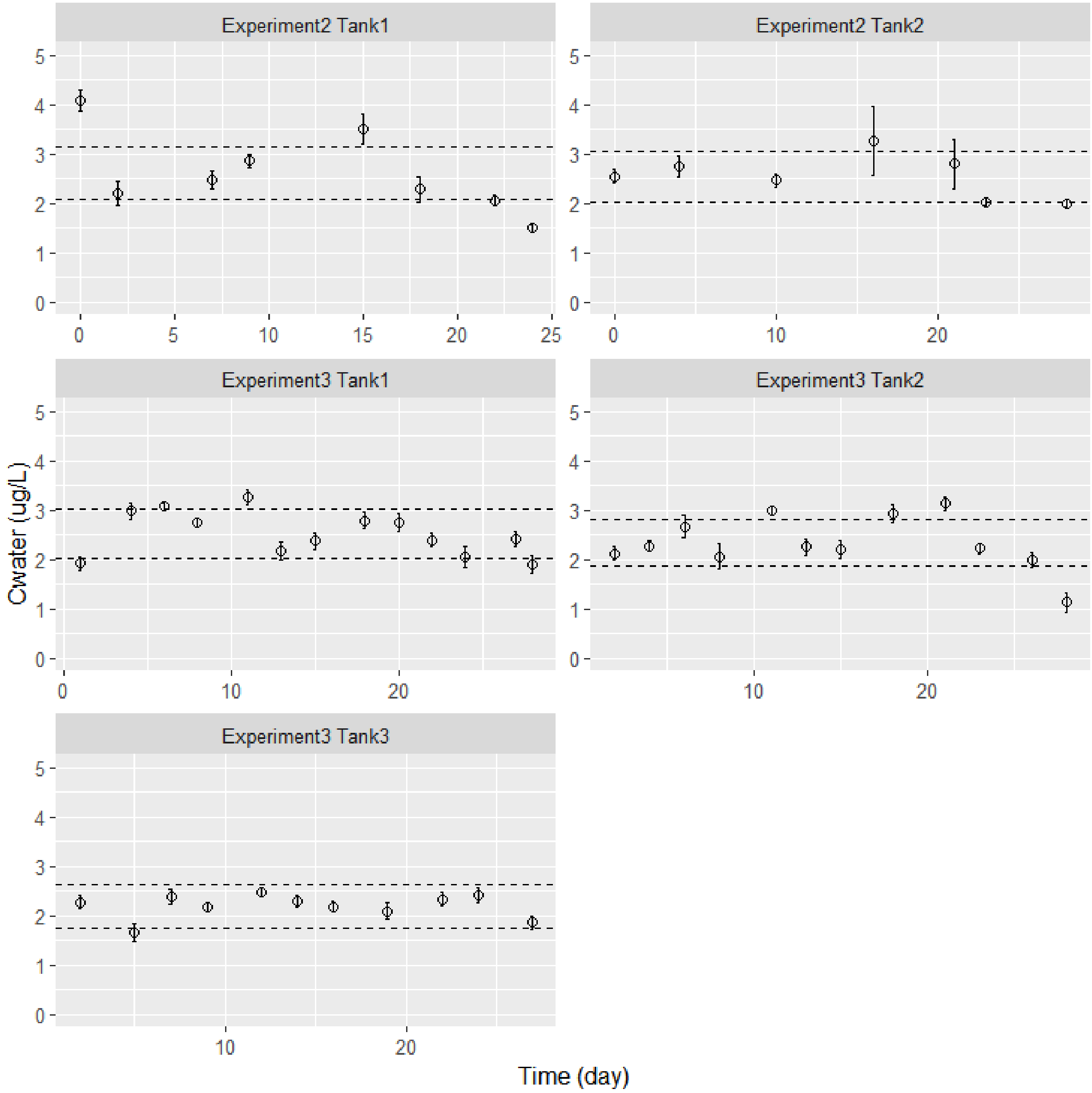

Figure S7-1: Mean aqueous phenanthrene concentration (µg/L), including the standard deviation, during the uptake phase for (a) Experiment 2 Tank 1; (b) Experiment 2 Tank 2; (c) The dashed (--) lines represent the ± 20% of the mean measured values during the uptake phase, according to the OECD 305 test validity criteria.

Carlson et al. [11] also attempted to investigate biotransformation enzyme induction in the test fish as a result of exposure to PAHs (Table S7-2). For example, one experiment (EXP#2Tank#1) exposed the fish to PHE and β-napthoflavone, a known potent inducer of CYP enzymes in fish. AHH activity (pM 3-OH-BaP)/(mg protein/min) as reported in Table 18 of Carlson et al. [11] are summarized in Table S7-2 below for the various experiments including PHE. Although AHH activity is not always significantly different from the control fish, there is a possible inverse relationship between AHH activity and reported Day 28 BCF for Exp#3 Tank#3. However, as stated by the authors of this study: “statements about the relationship between AHH activity and bioaccumulation are difficult to make. The rather large errors involved in the AHH activity measurements using the reported fluorimetric procedure and the previously mentioned errors in bioconcentration factor values make conclusions tenuous.”

Table S7-2: AHH Activity as reported in Table 18 of Carlson et al. [11] versus BCF at Day 28. The mean and standard deviation reported for controls was 0.53±0.21 (pM 3-OH-BaP)/(mg protein/min)

EXP #	TANK #	Compounds Present	AHH Activity* Day 7	AHH Activity* Day 14	AHH Activity* Day 28
2	1	Phenanthrene<br>β-napthoflavone	1.0±0.4	1.0±0.5	0.69±0.04
2	2	Phenanthrene	0.31±0.02	0.4±0.1	0.44±0.02
3	1	Phenanthrene<br>9-chlorophenanthrene	0.37±0.08	1.5±0.2	1.3±0.3
3	2	Phenanthrene	0.7±0.3	0.7±0.3	0.5±0.2
3	3	Phenanthrene<br>Dibenzofuran<br>Fluorene<br>1-methylphenanthrene<br>Fluoranthene<br>Pyrene	1.5±0.8	1.4±0.1	1.0±0.5

* (pM 3-OH-BaP)/(mg protein/min); see Carlson et al. 1979 (Table 18) for additional details

Application of the bcmfR Tool

The BCF data from the Carlson et al. [9] study were reanalyzed with the bcmfR Tool version 0.4-18 recommended by the OECD 305 guidelines. This tool allows for water and fish concentration data from both the uptake and depuration phase to be taken into consideration so that a kinetic BCFs, gill uptake rate constant (k1, L/kg/d) and total elimination rate constant (kT, 1/d) can be estimated, i.e., BCF = k1/kT [Figure S7-2]. The rate constants are fitted using three different statistical approaches, (i) an untransformed run, (ii) a ln-transformed run and (iii) box-cox transformed run and output includes standard errors and 95% confidence intervals. The best fit is determined statistically through the Shapiro-Wilk Normality test and the Runs test.

Note that application of the bcmfR tool does not alleviate concerns related to the accuracy of the reported water and fish concentration data but simply provides a more robust calculation of BCF compared to a concentration ratio from a single time point. The best fit uptake and elimination rate constants and 5% lipid standardized kinetic BCFs and 95% CI obtained from this reanalysis are summarized in Table S7-3.

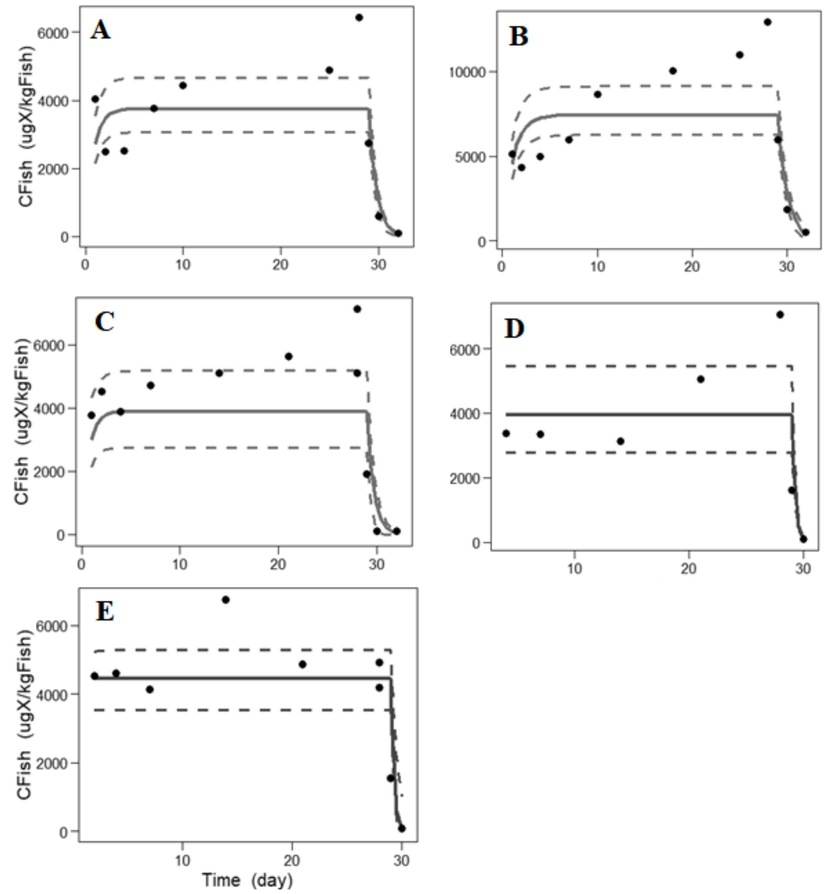

Figure S7-2: Concentration of PHE in fish (µg/kg) during uptake and depuration in (A) Experiment2 Tank1, (B) Experiment2 Tank2, (C) Experiment3 Tank1, (D) Experiment3 Tank2, (E) Experiment3 Tank3. Solid line represents the best model fit using the bcmfR tool for BCF calculation; standard error around the best fit is indicated with a dashed line (- -).

As shown in Figure S7-2, the best fit uptake curves indicate that steady-state is approached within a few days even though the reported concentrations of PHE in fish in four out of five studies continue to increase over the 28 day exposure period. Another challenge with fitting these data is the lack of frequent measurements taken at very early time points. This issue is most evident in the uptake curves for Experiment3 Tank2 (Panel D) and Experiment 3 Tank3 (Panel E). The lower BCFs derived from using the bcmfR tool are in part due to the fact that the model analysis must take into account the rapid reduction in tissue concentrations that is observed during the depuration phase when exposures are terminated as shown in Figure S7-2 for all experiments.

Gill uptake rate constants are expected to vary as a function of fish size, dissolved oxygen and water temperature as these are the key parameters influencing gill ventilation rate in laboratory BCF experiments. Because the fish and experimental conditions in Carlson et al. were very similar across all experiments, the four-fold range in estimated uptake rate constants is difficult to rationalize. Although fish weights were not reported by Carlson et al., expected gill uptake rate constants for 50-100 mg fish (i.e., 5–6 week-old fathead minnows) are in the range of 1000–4000 L/kg/d [42, 43]. Based on these estimates, the best fit gill uptake rate constants in EXP#3 are the least plausible whereas all other estimates appear reasonable.

Table S7-3 Summary of bcmfR Tool application to Carlson et al. 1979 [9] PHE data

EXP #	TANK #	Mixture?	Best Fit k1 (L/kg/d)	95% CI of k1 (range)	Best Fit kT (1/d)	95% CI of kT (range)	Best Fit 5 % lipid standardized kinetic BCF*	95% CI of BCF (range)[+]	95% CI of BCF (range)[#]
2	1	PHE+	1829	1128-2531	1.28	1.02-1.54	1488	1096–1879	516-2458
2	2	PHE	2749	1732-3766	0.95	0.72-1.17	3826	2972–4679	2318-5332
3	1	PHE+	2273	545-4001	1.47	0.29-0.91	1880	865–2896	611-3149
3	2	PHE	5446	2297-8597	3.57	2.52-4.63	1956	1208–2704	821-3092
3	3	PHE+	7762	na	3.84	na	2299	1787–2811	1597-3001

* Lipid contents of fish ranged from 3.8 to 4.8% and hence ratios of k1/kT do not equal the 5% lipid standardized values

[+] 95% confidence interval calculated directly from bcmfR tool and only includes variability in fish tissue concentration and thus ignores the contribution from variation in water concentrations. [#]includes added uncertainty from variability in phenanthrene water concentrations.

Elimination processes including biotransformation are also a function of fish size and water temperature and accordingly the range in estimated total elimination rate constants are also difficult to rationalize. For example, the near identical total elimination rate constants between EXP#3Tank#2 (kT = 3.67/d) and EXP#3Tank#3 (3.84/d) cannot be reconciled with the AHH activity data (i.e., proxy for biotransformation capacity) reported in Table S7-3.

Taken together, the most obvious discrepancies in the Carlson et al. data are the two-fold difference in uptake rate constant and four-fold difference in total elimination rate constant between the two replicate PHE only experiments (EXP#2Tank#2, EXP#3Tank#2) for which the potential influence of other test substances on biotransformation is not a potential confounding factor. These results do not seem reasonable given experiments conducted using the exact same exposure regime and using a large number of the same fish species under the same experimental conditions and within the same lab. Such lack of reproducibility coupled with the unexplained variation and large confidence intervals around the toxicokinetic parameters indicates BCFs derived from this study should be judged as unreliable. A further rationale to exclude these data in decision making is that the 5% lipid standardized kinetic BCF of the two PHE only studies from the Carlson et al. study are estimated to be 3826 and 1956 L/kg, with overlapping 95% confidence intervals when the variability in the water concentrations are factored into uncertainty bounds. Thus, the experimental precision of BCF results obtained from this study are inadequate to provide a defensible basis for B assessment.

As noted above, a successful application of the bcmfR Tool implies nothing about accuracy of the underlying measurements. In other words, error in the reported water and/or tissue concentrations remain a concern and cannot be discounted based on the reanalysis presented above. For example, phenanthrene was determined using an outdated quantification approach based on photoionization detection. This method is likely more prone to interferences than current day GC-MS analysis. This concern is reflected in the spiked recoveries reported for tissue samples which ranged from 67 to 116% for phenanthrene (c.f. Table 8 in Carlson et al. [9]).

Equilibrium Partitioning (Fugacity Ratio) Analysis

As discussed in the Methods section of the main paper, fugacity ratios for BCF data provide a useful quality check since these values are theoretically constrained to less than or equal to one. Fugacity ratios of one imply equilibrium partitioning between the concentration of chemical in water and the fish and that biotransformation is a negligible elimination route. Fugacity ratios are approximately equal to the ratio of empirical BCF to the equilibrium partitioning-based (EQP) BCF. The EQP BCF can be estimated from the composition of the organism (lipids, proteins) and the corresponding biopartitioning coefficients, i.e.,

$$EQP\ BCF = K_{FW} = f_L \cdot K_{SlW} + f_P \cdot K_{PW} + f_W$$

where K_{FW} is the equilibrium fish-water partition coefficient, f_L, f_P and f_W are the lipid, protein and water contents of the fish and K_{SlW} and K_{PW} are storage lipid and structural protein-water partition coefficients. EQP BCFs for the Carlson et al. studies using reported lipid contents, an assumed protein content of 15% and the ppLFER-derived partitioning data at 25 ^{o}C in Section S2 are compared to the BCFs as originally reported in Table S7-4.

Table S7-4. Comparison of BCFs as originally reported and estimated by the bcmfR Tool with estimated EQP BCFs (fugacity ratios)

EXP #	TANK #	Reported Lipid Content (%)	EQP BCF (L/kg)	BCF as originally reported (L/kg)	BCF ratio (Fugacity ratio)	Best Fit 5 % lipid standardized kinetic BCF	BCF ratio (Fugacity ratio)
2	1	4.8	3380	2500±1300	0.74	1487.6	0.44
2	2	3.8	2735	5100 ±1600	**1.86**	3825.7	**1.40**
3	1	4.1	2930	2000±600<br>2800±750	0.68<br>**0.96**	1880.4	0.64
3	2	4.3	3055	3100±1000<br>3000±1000	**1.02**<br>**0.98**	1956.3	0.64
3	3	4.4	3120	1900±500<br>2200±400	0.61<br>0.71	2299.2	0.74

Given the expected susceptibility of phenanthrene to biotransformation by fish (see section 3.2 below) fugacity ratios below unity are expected. As shown in Table S7-4, the BCF originally reported for EXP#2Tank#2 of 5100 L/kg is suspect (i.e., fugacity ratio > 1) followed by the BCFs reported for EXP#3Tank#2. The kinetic BCF based on bcmfR Tool output for EXP#2Tank#2 also corresponds to a fugacity ratio > 1 and is therefore also flagged as likely erroneous. The EQP (fugacity ratio) analysis based on the BCFs as originally reported implies error in the reported water and/or fish concentrations from half of the experiments (i.e., fugacity ratios ~ or > 1). With respect to the kinetics BCFs estimated by the bcmfR Tool, the fugacity ratios cannot be interpreted without simultaneously considering the reasonableness of the estimated uptake and elimination rate constants.

This theoretical evaluation, taken together with the previous discussion, as well as results of other fish BCF studies captured in Table 1 of the main paper, provides a compelling weight of evidence assessment to conclude the Carlson et al. BCF data are unreliable for regulatory B assessment and decision-making.

Section S8. Fugacity ratio analysis of Khairy et al. 2014 BAFs

We originally included 19 field BAFs from the Khairy et al. study for the BAT assessment of PHE. The BAFs from Khairy et al. [23] are reported in units of L-freely dissolved water / kg-lipid fish and are very high. The lipid normalized BAFs were converted to wet weight BAFs but were still very high (>> 5000 L/kg). For chemicals like phenanthrene (log K_{OW} < 5) there should be little difference between total water concentrations and dissolved water concentrations under typical environmental conditions (e.g., total organic carbon content in the water ~ 2-3 mg/L).

The Khairy et al. study uses passive samplers to estimate the water concentrations. To reconcile the discrepancies in the BAFs (>> 5000 L/kg) and the TMF (<1) from this study we converted the calculated wet weight, freely dissolved water concentration BAFs to fugacity ratios by dividing by K_{BW}, including storage lipid, estimated phospholipid and protein phases. The BAFs and fugacity ratios are summarized in Table S8- 1. Fifteen of the 20 fugacity ratios are > 1 which indicates biomagnification is occurring. However, such inferences are completely counter to the overall food web TMF from this study indicating there is no biomagnification of phenanthrene in this food web (TMF = 0.34). The tissue concentrations in the reported BAFs and the reported TMF are the same, the only difference is in the water concentrations used to derive the BAFs. Furthermore, all existing evidence to-date shows that

phenanthrene does not biomagnify, which is also consistent with its physical-chemical properties. We believe there is an underlying source of error in the published BAFs perhaps due to errors in the water concentrations as derived from the passive samplers or some unit conversion error. We did not pursue this issue any further except to assign a "Critical Fail" to all of the BAFs reported in Khairy et al. [23] as entered in the BAT, but did not fail the TMF study as water concentrations are not used to calculate the TMF. We believe further scrutiny and analysis of the data reported in Khairy et al. [23] is warranted.

Table S8- 1: Summary of BAFs and fugacity ratios from the Khairy et al. [23] publication

**Organism**	**BAF (L/kg-lipid)**	**BAF (L/kg-ww)**	**Fugacity ratio**
Lepomis gibbosus	110000	2640	1.22
Fundulus diaphanus	26000	520	0.28
Lepomis macrochirus	73000	2409	0.84
Hybognathus regius	95000	3325	1.10
Esox americanus	52000	520	0.48
Fundulus diaphanus	140000	2800	1.51
Morone americana	99000	3960	1.16
Anguilla rostrata (11-12 cm)	170000	1700	1.57
Anguilla rostrata (28-110 cm)	93000	5580	1.12
Callinectes sapidus	1400000	32200	15.40
Hybognathus regius	430000	15050	4.99
Morone saxatilis (18-20 cm)	120000	1920	1.24
Morone americana	220000	8800	2.58
Morone saxatilis (9.6-10.4 cm)	100000	1000	0.92
Morone saxatilis (20-33 cm)	45000	742.5	0.47
Hybognathus regius	360000	12600	4.17
Dorosoma cepedianum	480000	5280	4.56
Fundulus heteroclitus	530000	5830	5.03
Menidia menidia (2.2-3.6 cm)	260000	7280	2.94
Menidia menidia (7.8-9.4 cm)	430000	10320	4.75

Literature Cited

[1] Ma Y-G, Lei YD, Xiao H, Wania F, Wang W-H. 2010. Critical review and recommended values for the physical-chemical property data of 15 polycyclic aromatic hydrocarbons at 25 °C. *Journal of Chemical & Engineering Data* 55:819-825.

[2] Ni N, Yalkowsky SH. 2003. Prediction of Setschenow constants. *International Journal of Pharmaceutics* 254:167-172.

[3] Abraham MH, Ibrahim A, Acree WE. 2007. Partition of compounds from gas to water and from gas to physiological saline at 310 K: Linear free energy relationships. *Fluid Phase Equilibria* 251:93-109.

[4] Geisler A, Endo S, Goss KU. 2012. Partitioning of organic chemicals to storage lipids: elucidating the dependence on fatty acid composition and temperature. *Environmental Science & Technology* 46:9519-9524.

[5] Beyer A, Wania F, Gouin T, Mackay D, Matthies M. 2002. Selecting internally consistent physicochemical properties of organic compounds. *Environmental Toxicology and Chemistry* 21:941-953.

[6] Schwarzenbach R, Gschwend P, Imboden D. 2003. *Environmental Organic Chemistry 2nd Edition*. John Wiley & Sons, Inc., Hoboken, NJ, USA.

[7] deBruyn AMH, Gobas FAPC. 2007. The sorptive capacity of animal protein. *Environmental Toxicology and Chemistry* 26:1803-1808.

[8] Mackintosh CE, Maldonado J, Hongwu J, Hoover N, Chong A, Ikonomou MG, Gobas FAPC. 2004. Distribution of phthalate esters in a marine aquatic food web: comparison to polychlorinated biphenyls. *Environmental Science & Technology* 38:2011-2020.

[9] Baussant T, Sanni S, Jonsson G, Skadsheim A, Borseth JF. 2001. Bioaccumulation of polycyclic aromatic compounds: 1. Bioconcentration in two marine species and in semipermeable membrane devices during chronic exposure to dispersed crude oil. *Environmental Toxicology and Chemistry* 20:1175-1184.

[10] Baussant T, Sanni S, Skadsheim A, Jonsson G, Borseth JF, Gaudebert B. 2001. Bioaccumulation of polycyclic aromatic compounds: 2. Modeling bioaccumulation in marine organisms chronically exposed to dispersed oil. *Environmental Toxicology and Chemistry* 20:1185-1195.

[11] Carlson R, Oyler A, Gerhart E, Caple R, Welch K, Kopperman H, Bodenner D, Swanson D. 1979. Implications to the aquatic environment of polynuclear aromatic hydrocarbons liberated from Northern Great Plains coal. United States Environmental Protection Agency, Duluth, MN, USA.

[12] Cheikyula JO, Koyama J, Uno S. 2008. Comparative study of bioconcentration and EROD activity induction in the Japanese flounder, red sea bream, and Java medaka exposed to polycyclic aromatic hydrocarbons. *Environmental Toxicology* 23:354-362.

[13] Freitag D, Ballhorn L, Geyer H, Korte F. 1985. Environmental hazard profile of organic chemicals: An experimental method for the assessment of the behaviour of organic chemicals in the ecosphere by means of simple laboratory tests with 14C labelled chemicals. *Chemosphere* 14:1589-1616.

[14] Jonsson G, Bechmann RK, Bamber SD, Baussant T. 2004. Bioconcentration, biotransformation, and elimination of polycyclic aromatic hydrocarbons in sheepshead minnows (*Cyprinodon variegatus*) exposed to contaminated seawater. *Environmental Toxicology and Chemistry* 23:1538-1548.

[15] Kobayashi J, Sakurai T, Mizukawa K, Kinoshita K, Ito N, Hashimoto S, Nakajima D, Kawai T, Imaizumi Y, Takada H, Suzuki N. 2013. Respiratory uptake kinetics of neutral hydrophobic organic chemicals in a marine benthic fish, Pseudopleuronectes yokohamae. *Chemosphere* 93:1479-1486.

[16] Li Y, Wang H, Xia X, Zhai Y, Lin H, Wen W, Wang Z. 2018. Dissolved organic matter affects both bioconcentration kinetics and steady-state concentrations of polycyclic aromatic hydrocarbons in zebrafish (*Danio rerio*). *The Science of the Total Environment* 639:648-656.

[17] Lo JC, Letinski DJ, Parkerton TF, Campbell DA, Gobas FAPC. 2016. In Vivo biotransformation rates of organic chemicals in fish: relationship with bioconcentration and biomagnification factors. *Environmental Science & Technology* 50:13299-13308.

[18] Wang H, Li Y, Xia X, Xiong X. 2018. Relationship between metabolic enzyme activities and bioaccumulation kinetics of PAHs in zebrafish (Danio rerio). *Journal of Environmental Sciences* 65:43-52.

[19] Wang H, Xia X, Liu R, Wang Z, Zhai Y, Lin H, Wen W, Li Y, Wang D, Yang Z, Muir DCG, Crittenden JC. 2019. Dietary uptake patterns affect bioaccumulation and biomagnification of hydrophobic organic compounds in fish. *Environmental Science & Technology* 53:4274-4284.

[20] Xia X, Li H, Yang Z, Zhang X, Wang H. 2015. How does predation affect the bioaccumulation of hydrophobic organic compounds in aquatic organisms? *Environmental Science & Technology* 49:4911-4920.

[21] Gobas FAPC, Lee Y-S, Lo JC, Parkerton TF, Letinski DJ. 2019. A toxicokinetic framework and analysis tool for interpreting Organisation for Economic Co-operation and Development Guideline 305 dietary bioaccumulation tests. *Environmental Toxicology and Chemistry*.

[22] Burkhard LP, Arnot JA, Embry MR, Farley KJ, Hoke RA, Kitano M, Leslie HA, Lotufo GR, Parkerton TF, Sappington KG, Tomy GT, Woodburn KB. 2012. Comparing laboratory and field measured bioaccumulation endpoints. *Integrated Environmental Assessment and Management* 8:17–31.

[23] Khairy MA, Weinstein MP, Lohmann R. 2014. Trophodynamic behavior of hydrophobic organic contaminants in the aquatic food web of a tidal river. *Environmental Science & Technology* 48:12533-12542.

[24] Takeuchi I, Miyoshi N, Mizukawa K, Takada H, Ikemoto T, Omori K, Tsuchiya K. 2009. Biomagnification profiles of polycyclic aromatic hydrocarbons, alkylphenols and polychlorinated biphenyls in Tokyo Bay elucidated by δ13C and δ15N isotope ratios as guides to trophic web structure. *Marine Pollution Bulletin* 58:663-671.

[25] Ke R, Li J, Qiao M, Xu Y, Wang Z. 2007. Using semipermeable membrane devices, bioassays, and chemical analysis for evaluation of bioavailable polycyclic aromatic hydrocarbons in water. *Archives of Environmental Contamination and Toxicology* 53:313-320.

[26] Nfon E, Cousins IT, Broman D. 2008. Biomagnification of organic pollutants in benthic and pelagic marine food chains from the Baltic Sea. *The Science of the Total Environment* 397:190-204.

[27] Moermond CTA, Traas TP, Roessink I, Veltman K, Hendriks AJ, Koelmans AA. 2007. Modeling decreased food chain accumulation of PAHs due to strong sorption to carbonaceous materials and metabolic transformation. *Environmental Science & Technology* 41:6185-6191.

[28] Petersen GI, Kristensen P. 1998. Bioaccumulation of lipophilic substances in fish early life stages. *Environmental Toxicology and Chemistry* 17:1385-1395.

[29] Landrum PF. 1988. Toxicokinetics of organic xenobiotics in the amphipod, *Pontoporeia hoyi*: role of physiological and environmental variables. *Aquatic Toxicology* 12:245-271.

[30] Landrum PF, Lotufo GR, Gossiaux DC, Gedeon ML, Lee JH. 2003. Bioaccumulation and critical body residue of PAHs in the amphipod, Diporeia spp: additional evidence to support toxicity additivity for PAH mixtures. *Chemosphere* 51:481-489.

[31] Frank AP, Landrum PF, Eadie BJ. 1986. Polycyclic aromatic hydrocarbon rates of uptake, depuration, and biotransformation by Lake Michigan *Stylodrilus heringianus*. *Chemosphere* 15:317-330.

[32] Jensen LK, Honkanen JO, Jæger I, Carroll J. 2012. Bioaccumulation of phenanthrene and benzo[a]pyrene in Calanus finmarchicus. *Ecotoxicology and Environmental Safety* 78:225-231.

[33] Agersted MD, Moller EF, Gustavson K. 2018. Bioaccumulation of oil compounds in the high-Arctic copepod *Calanus hyperboreus*. *Aquatic toxicology (Amsterdam, Netherlands)* 195:8-14.

[34] Cailleaud K, Budzinski H, Le Menach K, Souissi S, Forget-Leray J. 2009. Uptake and elimination of hydrophobic organic contaminants in estuarine copepods: an experimental study. *Environmental Toxicology and Chemistry* 28:239-246.

[35] Southworth GR, Beauchamp JJ, Schmieder PK. 1978. Bioaccumulation potential of polycyclic aromatic hydrocarbons in Daphnia pulex. *Water Research* 12:973-977.

[36] Landrum PF, Poore R. 1988. Toxicokinetics of Selected Xenobiotics in Hexagenia Limbata. *Journal of Great Lakes Research* 14:427-437.

[37] Cavaletto J, Nelepa T, Dermott R, Gardner W, Quigley M, Lang G. 2011. Seasonal variation of lipid composition weight, and length in juvenile Diporeia spp (Amphipoda) from lakes Michigan and Ontario. *Canadian Journal of Fisheries and Aquatic Sciences* 53:2044-2051.

[38] Wan Y, Jin X, Hu J, Jin F. 2007. Trophic dilution of polycyclic aromatic hydrocarbons (PAHs) in a marine food web from Bohai Bay, north China. *Environmental Science & Technology* 41:3109-3114.

[39] Wang D-Q, Yu Y-X, Zhang X-Y, Zhang S-H, Pang Y-P, Zhang X-L, Yu Z-Q, Wu M-H, Fu J-M. 2012. Polycyclic aromatic hydrocarbons and organochlorine pesticides in fish from Taihu Lake: Their levels, sources, and biomagnification. *Ecotoxicology and Environmental Safety* 82:63-70.

[40] Qadeer A, Liu M, Yang J, Liu X, Khalil SK, Huang Y, Habibullah-Al-Mamun M, Gao D, Yang Y. 2019. Trophodynamics and parabolic behaviors of polycyclic aromatic hydrocarbons in an urbanized lake food web, Shanghai. *Ecotoxicology and Environmental Safety* 178:17-24.

[41] OECD. 2012. OECD Guidelines for Testing Chemicals. Test No. 305: Bioaccumulation in Fish: Aqueous and Dietary Exposure. Organization for Economic Co-operation and Development, Paris.

[42] Arnot JA, Gobas FAPC. 2004. A Food Web Bioaccumulation Model for Organic Chemicals in Aquatic Ecosystems. *Environmental Toxicology and Chemistry* 23:2343-2355.

[43] Crookes M, Brooke D. 2010. Estimation of fish bioconcentration factor (BCF) from depuration data. Environment Agency, Bristol, UK.
